# The shift to 3D growth during embryogenesis of kelp species, atlas of cell division and differentiation of *Saccharina latissima*

**DOI:** 10.1242/dev.201519

**Published:** 2023-10-26

**Authors:** Ioannis Theodorou, Bénédicte Charrier

**Affiliations:** ^1^ Laboratory of Integrative Marine Models, Station Biologique de Roscoff, UMR8227, CNRS, Sorbonne University, Place Georges Teissier, 29680 Roscoff, France; ^2^ Plant Sciences Department, Faculty of Biosciences, Norwegian University of Life Sciences, NO-1432 Ås, Norway

**Keywords:** 3D growth, Kelp, Meristoderm, Cell differentiation, Tissue thickening, Cell division pattern, Embryogenesis, Asymmetrical cell division

## Abstract

In most organisms, 3D growth takes place at the onset of embryogenesis. In some brown algae, 3D growth occurs later in development, when the organism consists of several hundred cells. We studied the cellular events that take place when 3D growth is established in the embryo of the brown alga *Saccharina*, a kelp species. Semi-thin sections, taken from where growth shifts from 2D to 3D, show that 3D growth first initiates from symmetrical cell division in the monolayered lamina, and then is enhanced through a series of asymmetrical cell divisions in a peripheral monolayer of cells called the meristoderm. Then, daughter cells rapidly differentiate into cortical and medullary cells, characterised by their position, size and shape. In essence, 3D growth in kelps is based on a series of differentiation steps that occur rapidly after the initiation of a bilayered lamina, followed by further growth of the established differentiated tissues. Our study depicts the cellular landscape necessary to study cell-fate programming in the context of a novel mode of 3D growth in an organism phylogenetically distant from plants and animals.

## Teaser

**Summary:** A longitudinal, mediolateral wave of symmetrical and asymmetrical cell divisions and a short-range gradient of cell differentiation initiates and reinforces 3D growth during brown alga embryogenesis, ensuring body thickening up to the adult stage.

## INTRODUCTION

In most metazoans, it takes three cell divisions to establish a mass of cells organised and interconnected in all three spatial dimensions (hereafter called 3D growth) (Gilbert and Barresi, 2016). In animal clades, which display very different cleavage patterns, including radial, spiral, bilateral and rotational patterns, this cleavage takes place as soon as the zygote is formed. Early embryonic cleavage occurs in most bilaterians, except mammals, birds, reptiles and fishes, in which 3D growth emerges by cell migration to form a primitive streak from a pre-established 2D cell layer. Insects are a particular case in which 3D growth is established by the segmentation of an already voluminous multinucleate cell (coenocyte). The arrangement of cells during the early cleavage of animal embryos sets up the embryo axes and determines the future adult body plan (Hasley et al., 2017). Therefore, in most metazoans, the establishment of 3D growth by cell division (as opposed to cell migration) occurs immediately after fertilisation and involves all cells, which divide almost synchronously in a different axis at each step.

Plant cells are surrounded by a cell wall, making 3D-growth mechanisms necessarily more constrained mechanically than in animals. Although, in contrast to animals, plant organisms grow continuously, they initiate 3D growth as fast as most animals do. Angiosperms (e.g. *Arabidopsis*) follow exactly the same order of cell divisions as the radial cleavage pattern observed in metazoans (i.e. two perpendicular meridional divisions followed by an equatorial division) (Moukhtar et al., 2019). Somatic embryogenesis shows that, although positional information most likely predominates, remote chemical information is likely involved (Smertenko and Bozhkov, 2014) and indeed the initial cell division in the *Arabidopsis* zygote has been shown to be under the control of maternal auxin (Robert et al., 2018). 3D growth is strikingly different in gymnosperms, in which the zygote undergoes three cycles of mitosis before cytokinesis occurs (coenocyte). It nevertheless results in two superimposed rows of four cells leading to a proper embryo and a suspensor, respectively (Cairney and Pullman, 2007). This pattern can be likened to segmentation of a multinucleate mother cell, as observed in insects (regardless of the presence of the cell wall/membrane and of the number of nuclei) (Gilbert and Barresi, 2016).

Brown algae are an evolutionarily recent multicellular group that evolved independently from animals and plants (Baldauf, 2003; Derelle et al., 2016; Bringloe et al., 2020). Along their evolutionary trajectory, they convergently developed 3D tissues though different mechanisms, implying changes in the orientation of cell division in specific locations of the embryo. Interestingly, different modes of 3D growth exist in this single clade. For example, growth can be diffuse, whereby all cells divide and expand, or can be located in very precise positions within the thallus, in tissues reminiscent of the meristems of land plants (Charrier et al., 2012). Also, 3D growth can be established at the onset of embryogenesis, as in animals and plants, or later when the embryo is already made of several cells organised in a linear stack or a monolayered lamina. As a result, 3D growth produces thick, multilayered tissues.

**Fig. 1.**
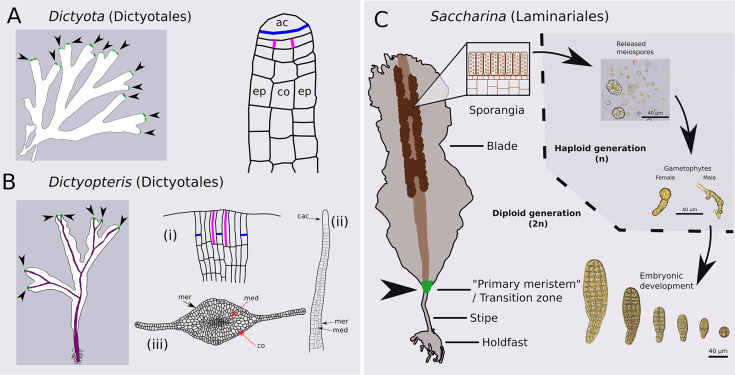
**Main growth modes in brown algae.** (A) Apical growth in *Dictyota dichotoma* (Phaeophyceae, Dictyotales), which is devoid of epidermal meristem (meristoderm). Left: Green areas and arrowheads indicate apical meristems (AM) at the apex of branches. Right: Sagittal section through the AM*,* the epidermal (ep) and cortical (co) cells derive from a series of anticlinal (ac; blue) and periclinal (magenta) cell divisions (Katsaros and Galatis, 1985). (B) Apical growth with meristoderm in *Dictyopteris membranacea* (Phaeophyceae, Dictyotales). Left: Green areas and arrowheads indicate the AM; the midrib, where the meristoderm resides, is shown in purple. (i) Frontal section of AM. Asymmetrical transverse divisions (blue) support apical growth, and longitudinal divisions (magenta) maintain the AM and some medio-lateral expansion (Katsaros and Galatis, 1988). (ii) Sagittal section of an apex. Cell layers are added below the central apical cell (cac) where the meristoderm (mer) and medulla (med) are first established. (iii) Transverse section below the apical region. The peripheral activity in the meristoderm establishes and maintains the cortex (co). (C) *Saccharina latissima* intercalary growth with an active meristoderm (green area and arrowhead) between the blade and stipe. The meristoderm in the transition zone (TZ) gives rise to the apico-basal, medio-lateral and thickening growth of mature specimen. Meiospores are produced in sporangia distal to the TZ. Released meiospores germinate into male or female gametophytes. Embryonic development starts with the polarisation of a spherical zygote. (A,B) Redrawn from Katsaros and Galatis (1985, 1988) with the permission of the main author. (C) Based on schematics from Theodorou and Charrier (2021).

Regarding the location of 3D growth, most parenchymatous brown algae have an external epidermal meristem, called the meristoderm (a term coined by the French phycologist C. Sauvageau; Sauvageau, 1918) that underlies most 3D growth (Fritsch, 1945) (Fig. 1). In addition, several orders of brown algae have an apical meristem (Katsaros, 1995), which, as in plants, is responsible for the growth of the thallus along the main longitudinal axis (usually apico-basal) (Clayton et al., 1985; Klemm and Hallam, 1987; Linardić and Braybrook, 2017). However, unlike plants, this apical meristem usually leaves the formation of thick tissues and lateral growth to the meristoderm.

For example, the brown algal order Dictyotales is characterised by a main apical meristem with one or more apical cells (Fig. 1A,B). Two case examples, *Dictyota* and *Dictyopteris*, base their apico-basal growth on an apical meristem, but they have different strategies regarding lateral growth and thickening. In *Dictyota*, the outer and inner cell divisions take place in the apical meristem, but *Dictyopteris* relies on the mitotic activity of the meristoderm. Similarly to *Dictyopteris*, Fucales such as *Sargassum* have an apical meristem and a meristoderm (Linardić and Braybrook, 2017). Interestingly, growth in the apical meristem of *Sargassum* takes place in a tetrahedral, continuously dividing apical cell, similar to the pattern observed in the leafy shoot of the moss *Physcomitrium* (Harrison et al., 2009).

Kelps, by contrast, adopt a completely different mode of 3D growth. Their embryo grows first as a monolayer cell sheet for several days before thickening, which only then adds a new axis of growth. Devoid of an apical meristem, kelps differentiate a transition zone (TZ) at an intercalary position, which ensures the growth of the blade (lamina) in the upper part and of the stipe in the lower part (Fig. 1C). The literature often identifies this intercalary zone as a meristem (Sykes, 1908; Steinbiss and Schmitz, 1974; Charrier et al., 2012; Coppin et al., 2020; Zhu et al., 2021). However, according to Smith (1939) and his work on several kelp species, this is not the case. Kelps base their 3D growth on a meristoderm (reviewed by Theodorou and Charrier, 2021), which is more active at the TZ (Smith, 1939; Fritsch, 1945), and in which two events take place: (1) periclinal divisions for the radial development of internal tissues (i.e. thickening) and (2) anticlinal cell divisions for the circumferential expansion of the meristoderm as the tissue expands in thickness. Cells that derive from periclinal divisions eventually differentiate into cortical cells, but can also add cell layers to the meristoderm in mature algae (Smith, 1939). Thereafter, the cortical cells gradually differentiate into medullary cells, which constitute the transport elements for photo-assimilates and nutrients in kelps (Schmitz et al., 1972; Lüning et al., 1973; Knoblauch et al., 2016). In general, there is a relatively good understanding of the growth of the mature stages from a descriptive point of view (for a review, see Theodorou and Charrier, 2021), enhanced by the recent growing interest in kelp cultivation and breeding (Hwang et al., 2019; Li et al., 2020; Forbord et al., 2020; Monteiro et al., 2021; Liesner et al., 2022). However, little is known about when and how 3D growth is established in the embryonic stages of kelps. Although our predecessors from the beginning of 20th century provided detailed drawings of what tissues look like prior to and after the formation of 3D tissues (Drew, 1910; Yendo, 1911), the transition from one stage to the other has not been described in detail. This gap precludes progress in the identification and study of factors involved in the spatiotemporal control of cell division activity and orientation during the formation of these 3D tissues.

Therefore, in this study, we monitored the early development of the kelp *Saccharina latissima*, with a special focus on the cellular steps that are responsible for the initiation of embryo thickening (named polystromatisation). We used this species because it is one of the simplest kelp species at the morphological level (Starko et al., 2019), in addition to having great economical (Theodorou et al., 2022), ecological (Christie et al., 2019) and evolutionary (Neiva et al., 2018) interests. In this study, we shed light on the cellular events prior to, during and after the shift from 2D to 3D growth in all three spatial dimensions. It lays the groundwork for future investigations of the molecular and mechanical control of 3D growth in kelps and other brown algal species.

## RESULTS

### The *Saccharina* embryo develops 3D tissues in three distinct, time-spaced steps

**Fig. 2.**
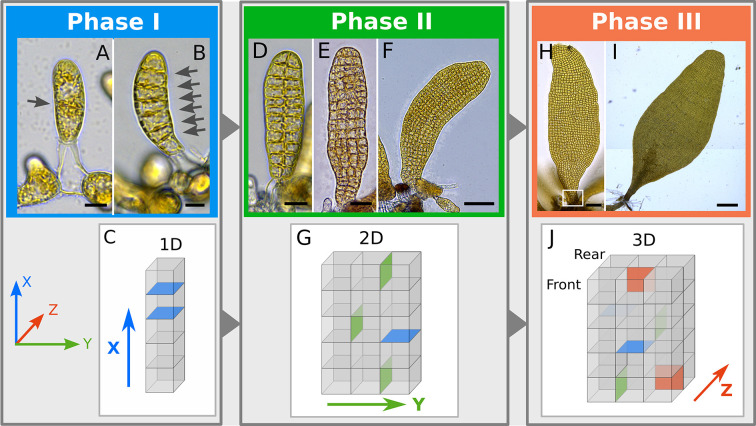
**Developmental phases during *Saccharina* embryogenesis.** Top: Bright-field images from developing embryos. Bottom: Schematics indicating the addition of division planes and growth axes. Phase I: (A) Zygote polarisation and first unequal division (arrow) 24 h after zygote polarisation (azp). (B) A series of anticlinal divisions (arrows) (60 h azp). (C) Simplified depiction of cell division during Phase I showing the transverse anticlinal cell division plane and the apico-basal axis (arrow) (*x*-axis, in blue). Phase II: (D) First longitudinal divisions, 132 h azp. (E) Advanced stage, 240 h azp. (F) Last stage of Phase II 324 h azp. (G) Longitudinal anticlinal (green) along with transverse (blue) divisions, forming a monostroma made of cells aligned in rows and columns (grid). Presence of a secondary growth axis is indicated (*y*-axis, green arrow). Phase III: (H) First periclinal divisions at the base of the embryo 16 days azp (white box). (I) Further periclinal divisions and addition of cell layers (dark brown region) along the *x*-axis, 20 days azp. (J) Polystroma during Phase III. Periclinal cell divisions (red) establish a third growth axis (*z*-axis, arrow) in the developing embryo. Axial system indicated in lower left corner. Scale bars: 10 μm (A,B); 15 μm (D); 25 μm (E); 70 μm (F); 90 μm (H); 300 μm (I). Representative images from 20 specimens. We designate as *x*-axis the first growth axis, then the *y*- and *z*- are the second and third growth axes respectively. Panels D, E and H show *z*-projections through Stack focuser on FIJI.

We initiated our study by dividing *Saccharina* embryogenesis into three phases based on the growth axis of its tissues and on the corresponding orientation of cell divisions (Table 1). Phase I (Fig. 2A,B) starts with the establishment of the main (apico-basal) growth axis (*x*-axis). This occurs with the initial elongation of the zygote seemingly induced by fertilisation. It is first followed by a first transverse anticlinal cell division, which appears unequal (Fig. 2A). Transverse anticlinal cell divisions are defined here as divisions perpendicular to the growth axis of the embryo (blue in Fig. 2C). Then, a series of transverse anticlinal divisions parallel to the first one reinforces the *x*-axis and the general anisotropic shape of the embryo (Fig. 2B). The second phase, Phase II, is initiated by the first longitudinal (parallel to the *x*-axis), anticlinal cell divisions (Fig. 2D), thereby establishing the second (medio-lateral) growth axis (Fig. 2D-F). Longitudinal anticlinal divisions are defined here as cell divisions parallel to the *x*-axis of the embryo and perpendicular to the surface (green in Fig. 2G). For about 14-16 days after zygote polarisation (azp) (Table 1), the embryo develops as a monolayer cell sheet (monostroma) (Fig. 2D-G). This monostroma is clearly visible on orthogonal views of confocal stacks of living embryos stained with Calcofluor (Fig. S1). In addition, because the mother and daughter cells are cuboid in shape, this first series of anticlinal (transverse and longitudinal) cell divisions leads to a cell arrangement resembling a grid, in which the cells are spatially arranged in rows and columns in contact with each other through four-way junctions (Fig. S1A). We consider the cells of Phase I and Phase II embryos to be ‘undifferentiated’ because, although photosynthetically active, they share a similar regular cuboidal shape, relatively low mitotic activity and a similar pattern of symmetrical cell division. As a result, the embryo resembles a monotonous monolayer lamina. Phase III starts when mature Phase II embryos (Fig. 2H) initiate the first tilting of the cell division plane on the *z*-axis (perpendicular to the *x*- and *y*-axes), thereby beginning embryo thickening (Fig. 2H-J). These cell divisions are defined as periclinal because they occur parallel to the largest surface of the embryo lamina. They are perpendicular to the anticlinal divisions (both transverse and longitudinal) (red in Fig. 2J). These three phases make up the early embryogenesis steps of *Saccharina*.

**Table 1.**
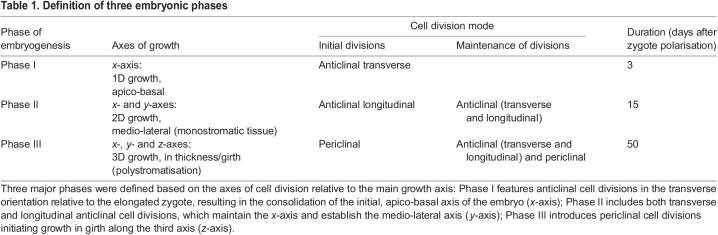
Definition of three embryonic phases

### The initiation of polystromatisation takes place at the base of the Phase II lamina

**Fig. 3.**
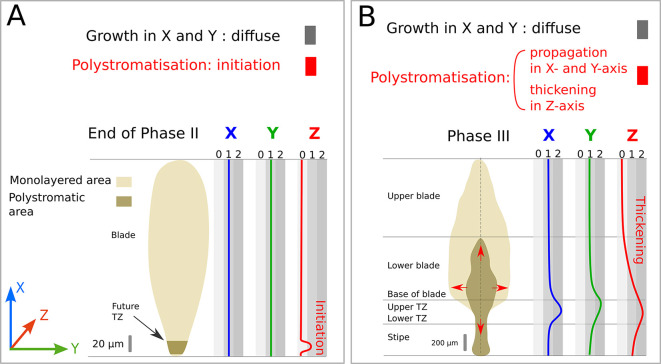
**Growth activity in the three spatial axes.** The relative growth rate (from 0: no growth to 1 or 2 levels of growth rates) in *x* (blue), *y* (green) and *z* (red) directions is depicted along the apico-basal axis at two different developmental stages (Phase II and Phase III). Monostromatic (light brown) and polystromatic regions (dark brown) are displayed for both stages. (A) Late Phase II: Polystromatisation is initiated in the basal region of the embryo (dark brown); the rest of the blade is still monostromatic (light brown). (B) Phase III: Polystromatisation progresses in three directions (*x*-, *y*-, *z*-axes) throughout the blade (red arrows). TZ, transition zone.

**Fig. 4.**
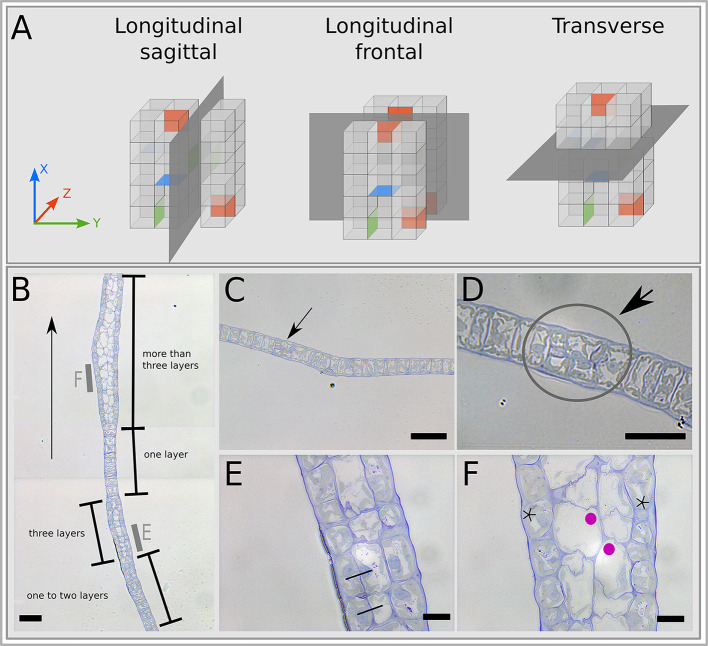
**Semi-thin sections of polystromatisation region.** (A) Schematics of the orientation of semi-thin sections based on our axis system as defined in Fig. 2. (B-F) Sagittal sections of 20-day-old embryos. (B) Paracentral sagittal section in the region where polystromatisation is initiated. Brackets indicate different states of the embryonic tissue along the *x*-axis (arrow indicates apico-basal direction). Note the asynchronous nature of polystromatisation. (C,D) First symmetrical divisions in a monostromatic region. The arrows and grey circle indicate the dividing cells. D is an enlarged view of C. (E) A three-layer region observed in B (grey bar). A middle layer is visible between two epidermal layers, corresponding to one of the two daughter cells following the division of one of the meristoderm layer (black lines connect the two daughter cells of two adjacent periclinal divisions). (F) Four-layer region observed in B (grey bar). The meristoderm (stars) and cortex (magenta dots) are distinguishable. The cortex is made up of large, angular transparent cells whereas the meristoderm cells are considerably smaller and cuboid. Note that these cortical cells can divide along the *x*-axis, as illustrated by the thin, anticlinal, transversal cell walls. Scale bars: 50 μm (B,C); 30 μm (D); 10 μm (E,F). Representative images from two specimens.

We termed the establishment of embryo thickening as polystromatisation, because it adds additional cell layers (stromata). When observed by bright-field microscopy, the monostromatic tissue of late Phase II embryos keeps growing through diffuse growth of all cells in *x* and *y* dimensions, whereas growth in *z* initiates at the base of the lamina (summarised in Fig. 3A). However, the precise location of growth initiation in the *z*-axis cannot be determined easily, making it difficult to track precisely. We noticed that, once initiated, polystromatisation spreads longitudinally and laterally (Fig. 3B, red arrows). However, this spreading is irregular, so that on sagittal sections (Fig. 4A), polystromatic laminae appear to be interspersed by monostromatic zones, as illustrated in Phase III embryos (Fig. 4B). Taking advantage of this property, we described how thickening of a monostromatic lamina occurs by preparing and observing several semi-thin sections from Phase III 20-day-old embryos (Fig. S2A; details on section replicates in Table S1) and focusing on areas where polystromatisation initiates. In these areas, we observed the series of cell division events underlying the transition of the monostroma to polystroma (multilayered tissue).

**Fig. 5.**
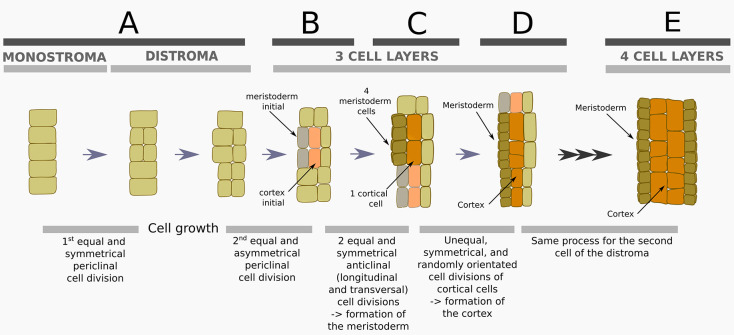
**Cellular patterning during polystromatisation.** Graphical representation of the events leading to the addition of stromata (polystromatisation) and emergence of meristoderm and cortex based on Fig. 4. (A) A distroma (bilayer) is established after symmetrical (same cell fate) and equal (in size) divisions of monostroma cells, without any prior cell growth. (B) After cell growth of the daughter cells, equal but asymmetrical divisions take place. The outer derivative is a meristodermal initial cell (mi); the inner cell is a cortical initial (ci). (C) Four meristoderm cells derive from two anticlinal divisions of mi perpendicular to each other; ci expands considerably in size forming a cortical cell. (D) Further anticlinal (longitudinal and transverse) divisions expand the meristoderm and cortex. However, the cortex shows random orientation of (un)equal and symmetrical divisions. (E) The same steps occur for both sister cells of distroma (A), either successively or at the same time, leading to four distinct cell layers in the new polystroma. Schematics are not drawn to scale.

Polystomatisation starts first with a periclinal division (Figs 4C,D and 5A). This division is equal and symmetrical, meaning that the two daughter cells are of similar size and fate. It is not preceded by cell expansion. Second, after cell growth, one of the sister cells divides periclinally into two cells of similar size (i.e. equal division; Figs 4E and 5B). Whereas the first division was symmetrical, this second division is asymmetrical, with daughter cells having different fates. The outer cell differentiates into a meristoderm initial (mi; grey in Fig. 5B) and the inner cell into a cortex initial (ci; pink in Fig. 5B). Third, the mi divides symmetrically anticlinally (both longitudinally and transversely) and produces small meristoderm cells at the surface of the lamina (Figs 4F and 5C; Fig. S3A), whereas ci enlarges before dividing along no specific axis (Figs 4F and 5D; Fig. S3B).

**Fig. 6.**
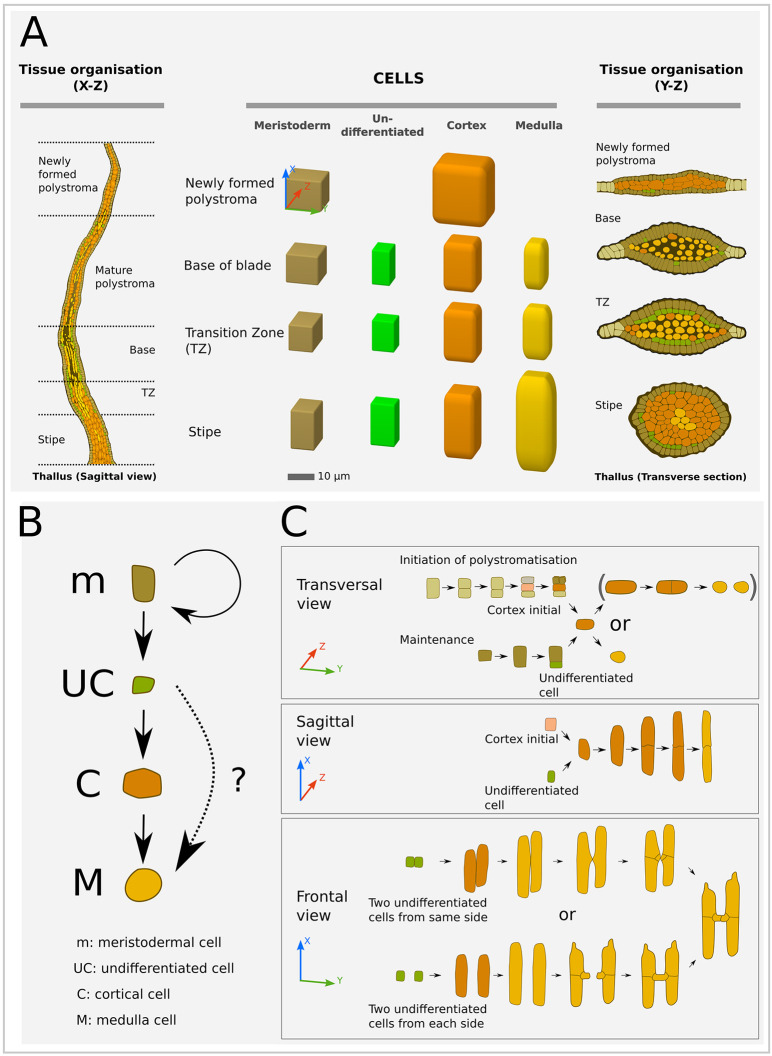
**Cell-type formation in *Saccharina* embryos.** (A) Cell types in relation to their position along the embryo. Left: Schematised sagittal section illustrating the different histogenetic zones along the apico-basal axis of the blade. Centre: Schematised shape and size of the four cell types: meristoderm, undifferentiated cells, cortex and medulla. The cell dimensions are shown according to the usual *xyz* spatial coordinates indicated in the top meristoderm cell. (see also data in Table S2 and box plots in Fig. S5). Note the increasingly rounded cell corners from meristoderm to medulla. Right: Schematics of corresponding transverse sections (same colour code). (B) Interpretation of the sequence of cell differentiation in polystromatic regions. Periclinal divisions in meristoderm (m) are asymmetrical and unequal in size. The largest cell retains its meristoderm identity, the smallest is an undifferentiated cell (UC). There are two possible pathways for a UC, either differentiation into a cortical (C) and then medullary (M) cell or directly into a medullary cell. The first pathway is observable as a gradient along the *z*-axis. This gradient is absent from the base of the blade where cortex is sparse, prompting our hypothesis for a second pathway. (C) Details of the differentiation of the cortical cells to the medullary cells in three different views (defined in Fig. 4A; spatial axes corresponding to the view are indicated). Note that the anticlinal cell division (*y*-axis) of the cortical cells in the top panel (transversal view) is rare (indicated by brackets). Frontal views depict the formation of branches (cross connections between medullary elements) through fusion of cell protrusions (Fig. S4D), as previously described by Killian (1911). Other authors (reviewed by Fritsch, 1945) report that the formation of cross connections can begin with horizontal extension of areas surrounding pit fields (brackets in Fig. S4E,H).

In parallel with this process, or subsequently, the second daughter cell of the initial division follows the same scheme of cell division and differentiation as the first one described above, establishing a second thickening layer from the initial monolayered lamina (Figs 4F and 5E). Therefore, at this four-cell-layer stage, called a newly formed polystroma, the blade is composed of two cell types: (1) one outer layer of small, plastid-rich cells that form an epidermal tissue with meristematic properties (meristoderm; Fig. S2B,C, Fig. S3A; one layer on each surface) and (2) an inner, thick layer made of wide, elongated transparent cells with big vacuoles that make up the cortex (Fig. 6A, top row; Figs S2C and S3B). Cell morphometrics are summarised in Table 2 and detailed in Table S2.

**Table 2.**
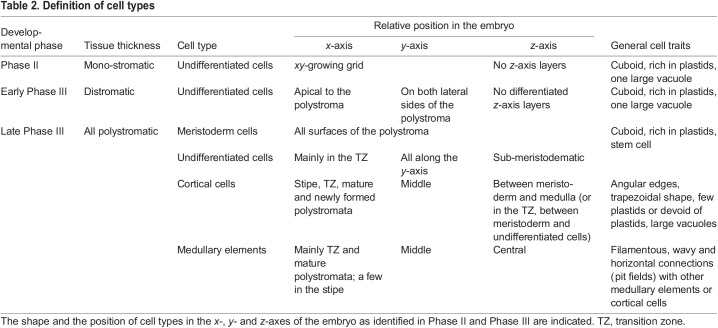
Definition of cell types

### Ongoing 3D growth strengthens in the TZ

The cell division and differentiation events described above remain located at the margins of the polystromatic tissue as the initial polystromatisation wave propagates in the *x* and *y* axes (Fig. 3B, red arrows). Approximately 60 days azp, all the monostroma is totally replaced by polystroma (Fig. S2D-H).

In the meantime, polystromatisation is reinforced throughout the whole thallus from the TZ, which begins at the base of the blade (Fig. 3B, red line). This TZ will become a distinct tissue located between the blade and the stipe (Smith, 1939; Fritsch, 1945; Theodorou and Charrier, 2021). In this tissue, growth in the *x* direction is enhanced both acropetally and basipetally (Fig. 3B, blue line), mainly as a result of cell divisions that ensure most of the further longitudinal growth of the embryo up to the adult stage. Slightly above this area, growth is enhanced along the *y*-axis, from the centre of the blade towards its ‘wings’ (edges), resulting in a broadening of the basal part of the blade (Fig. 3B, green line).

Altogether, the growth activity in the *Saccharina* embryo is finely orchestrated in the three spatial axes (Fig. 3A,B). This growth pattern ultimately results in a very anisotropic body shape, leading to a thick, elongated, flat lamina measuring up to 3 m in length, about 30 cm in width and ∼0.5 cm in thickness.

### Tissues start differentiating in the TZ

**Fig. 7.**
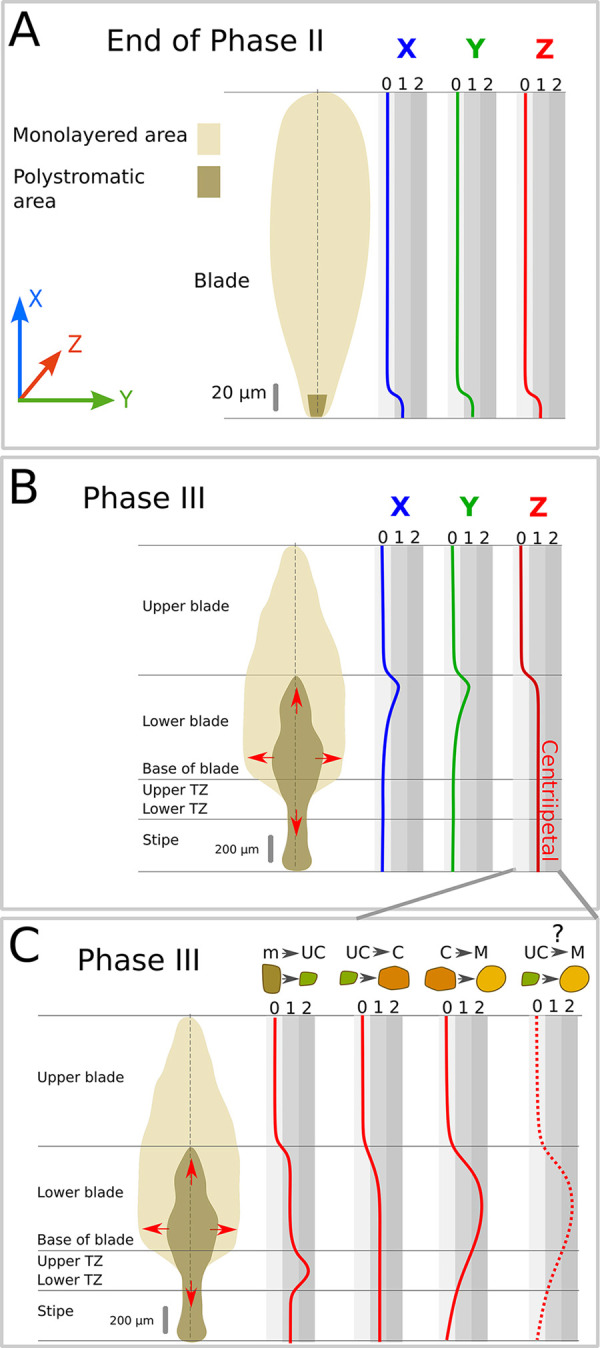
**Cell differentiation activity in the three spatial axes.** The relative rate of cell differentiation (from 0, indicating no cell differentiation, to 1 or 2 levels of differentiation rates) in the *x*- (blue), *y*- (green) and *z*- (red) axes is depicted along the apico-basal axis at two different developmental stages and through the median plane of the embryo (vertical dashed lines). Monostromatic (light brown) and polystromatic regions (dark brown) are displayed in the schematics of embryos. (A) Late Phase II: Differentiation is initiated at the basal region of the embryo at the same time and location as polystromatisation. (B) Phase III: Cell differentiation takes place as polystromatisation propagates. In the *x*- and *y*-axes, cell differentiation is observed at the front of the polystromatisation wave. In the *z*-axis, cell differentiation continues in the polystromatic region. (C) Individual steps of cell differentiation according to Fig. 6B. Undifferentiated cells are observed as soon as polystromatic tissue is formed and their multiplication is amplified in the transition zone (TZ) (first red curve on the left). These undifferentiated cells differentiate into cortical cells, which in turn differentiate into medullary cells mainly in the basal part of the blade where the medulla is abundant. Undifferentiated cells may potentially differentiate directly into medullary cells in this region (dotted red line on the far right). In contrast, medullary cell formation from cortical cell differentiation is rare in the stipe and basal part of the TZ, which are mainly composed of cortex (third red curve from the left). The red arrows indicate the direction of the propagation of polystromatisation (as in Fig. 3).

Concomitantly to cell growth, periclinal divisions are accompanied by cell differentiation in the three spatial axes. As with 3D growth, differentiation starts at the basal part of late Phase II embryos (future TZ) (Fig. 7A) and spreads throughout the thallus (Fig. 7B). We were able to observe a series of cell differentiation events on 20-day-old embryos starting from the cuboidal meristoderm cells that differentiated in the newly formed polystroma (Fig. 6A, brown cells). The first periclinal cell division is unequal and asymmetrical (Fig. 6B,C, transversal view). The outer peripheral cell is a new meristoderm cell and the inner cell is an ‘undifferentiated’ cell (UC; Fig. 6A-C, green cells; Fig. S4A). We called this inner daughter cell ‘undifferentiated’ because it corresponds to an intermediate stage before differentiation. The cortical cell (C) that differentiates from one UC gives rise either directly to one medullary cell (M) or divides periclinally (Fig. 6C, transversal view) or transversely (Fig. 6C, sagittal view; Fig. S4B) prior to differentiating into an M cell. Differentiation into mature M cells relies on at least two conspicuous processes. First, C cells elongate prior to differentiating into M cells (Fig. 6C, sagittal and frontal views) and, second, M cells make protrusions that promote intercellular connections (Fig. 6C, frontal view; Fig. S4D-H). This may be accompanied by modifications of the cell wall (Fritsch, 1945).

It should be noted that we found great variability in the shape and size of the cells of a given type, depending on their position along the *x*-axis of the embryo (Fig. 6A, centre; Fig. S5; Table S2). Briefly, cortical cells are wider in the newly formed, flat polystroma, and medulla cells are longer in the stipe than in the other regions of the embryo.

Below, we detail the diversity of cell types based on their shape and size and apparent rate of cell differentiation in different tissues positioned along the apico-basal axis of the Phase III embryos.

### In the blade of the embryo, medulla differentiates rapidly

**Fig. 8.**
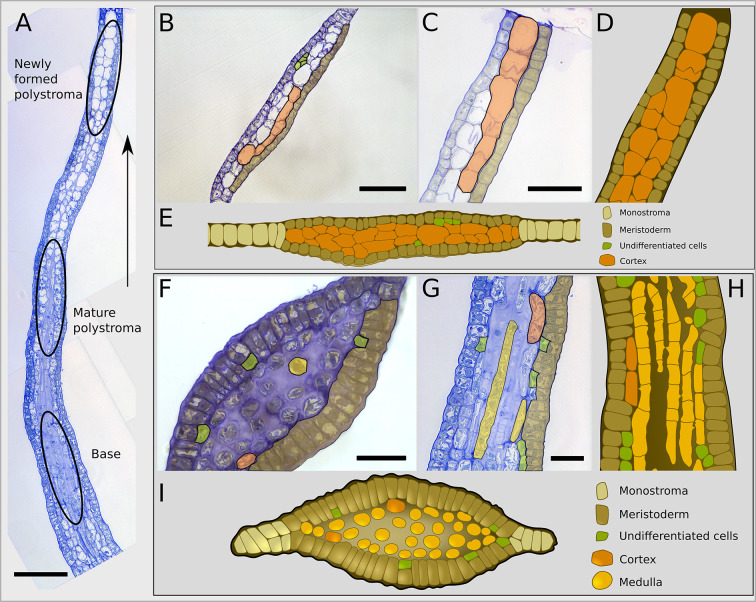
**Semi-thin longitudinal and transverse sections of blades in Phase III.** (A) Median sagittal section from the base of the blade to the newly formed polystroma, passing through the centre of the blade of a 20-day-old embryo. Polystromatisation progresses along the *x*-axis as described in Figs 3 and 7. Circled areas indicate the different positions in the polystromatic blade. The progression of polystromatisation can be observed in relative time, from the new polystroma to the base of the blade, where it is more advanced. (B-E) Transverse (B) and sagittal (C) sections of newly formed polystroma, with associated schematics (D,E). (F-I) Transverse (F) and sagittal (G) sections of the base of the blade, with associated schematics (H,I). Note that the contours of the cortical cells in the new polystroma are generally aligned with one or up to three meristodermal cells (A,C-E), which supports that they are derived from periclinal cell division of meristodermal cells (the latter may have divided anticlinally once or twice subsequently, giving three meristodermal cells for one cortical cell). No periclinal cell division of cortical cells could be observed in these newly formed and more mature polystromata. The approximate location of the sections are shown in Fig. S8. Scale bars: 110 µm (A); 50 μm (B,C); 30 μm (F,G). These representative images are from two specimens per orientation of section. In B,C,F,G, cell types are pseudocoloured as a semi-transparent overlay on bright-field images.

Whereas in the top part of the blade at the front of the polystromatisation wave, the newly formed polystroma is formed of only meristodermal and cortical cells (Fig. 8A, top; Fig. 8B-E), in the base of the blade the cortical tissue is reduced and is replaced by a large intercellular space characteristic of mature polystroma (Fig. 8A, bottom; Fig. 8F,G). Following this gradual replacement, we observed that cortical cells often divide perpendicularly to the *x*-axis (Fig. 6C, sagittal view; Fig. S4B) before mucilage deposition (observed also by Fritsch, 1945, p. 227). This mucilage deposition results in the thickening of the lateral cell walls that eventually separates the cells (Fig. 8H,I). These cells then differentiate into medullary cells (Figs 6B,C and 7C). Generally, medullary cells in the blade are filamentous, thinner than cortical cells, but of similar *x*-axis length (Fig. 6A, second row in the centre; Fig. 6C, frontal and sagittal views; Fig. 8G-I, Fig. S2E-G). They seem to expand primarily along the *x*-axis (Fig. 8G). They generally divide anticlinally, but periclinal divisions also occur, probably as a function of branching (Fig. S4C-G, arrows and brackets). Several connections are visible between two medullary cells (e.g. Fig. S4G,H, brackets; schematised in Fig. 6C, frontal view), and also between medullary and cortical cells (Fig. S4E,H, brackets). These connections, which appear to rely on the prior formation of protrusions, are probably pit fields that can resist changes in cell shape during cortex-to-medulla differentiation (Fig. S4C,D, arrows).

Finally, in all regions of the polystromatic blade, a few undifferentiated cells can be observed, seemingly the result of asymmetrical cell divisions of meristoderm cells (green cells schematised in Fig. 6 and shown in Fig. 8B,E-I), which eventually differentiate into cortical cells (Figs 6B,C and 7C).

### In the stipe of the embryo, the medulla differentiates slowly

**Fig. 9.**
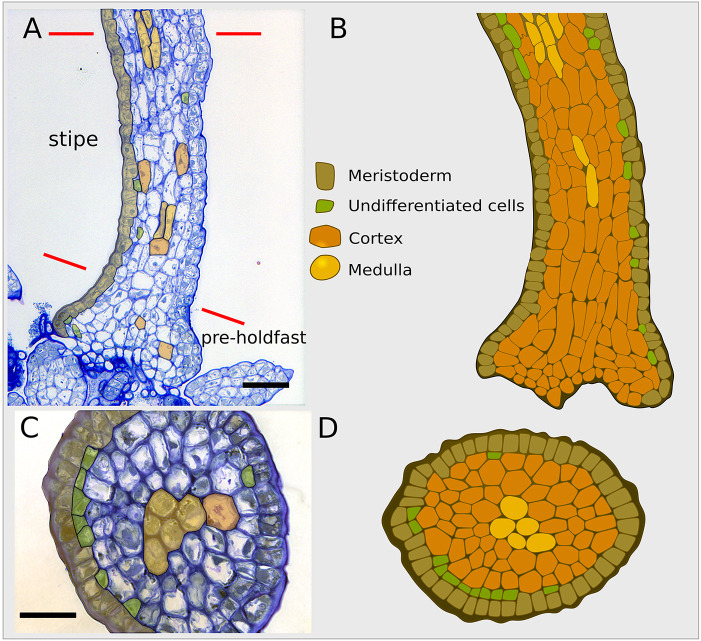
**Semi-thin longitudinal sagittal and transverse sections of a stipe in Phase III.** Compare these sections with Fig. 8. (A-C) Bright-field images of semi-thin sections. (A,C) Median sagittal section (A) and transverse section (C) of a 20-day-old embryo. The red lines delimit the stipe region. At the bottom of the stipe is the pre-holdfast. (B,D) Schematics of sagittal (B) and transverse (D) sections the stipe. Scale bars: 50 μm (A); 30 μm (C). Representative images from two specimens per orientation of section. In A,C, cell types are pseudocoloured as a semi-transparent overlay.

In general, the stipe consists of the same cell types and tissues as the polystromatic blade (Fig. 6A, bottom row). Its tissue organisation is more homogenous than in the blade and with no large intercellular spaces. Only a few medullary cells are present and are observed mostly in central positions in the upper part of the stipe near the TZ (Fig. 9A,B). Therefore, differentiation of cortical cells into medulla cells is slowed or hindered (Fig. 7C). This property is maintained in older algae, where the cortical tissue makes most of the stipe (Fig. S6).

In addition, in contrast to their blade counterparts, the few stipe medullary cells do not branch and are the longest cells among all the tissues observed in this study (Fig. 6A, bottom row; Fig. S5). This may be the result of longitudinal mechanical stresses applied to the stipe, which happens to be an elongated structure that can withstand pulling forces transmitted by the holdfast (the organ attaching the alga to the substratum; see pre-holdfast Fig. 9A). We observed that these differences persist for up to 4 months azp (Fig. S2H-K). Similarly, the cells of the cortex, which is the predominant tissue in the stipe (Fig. 9C,D), show differences from those of a newly formed polystroma cortex (as in Fig. 8B-E). Both have large rectangular transparent cells with angular edges (Fig. 6A), but their sizes differ. Their lengths along the *x*-axis are similar (24.93 μm±8.28 μm for the stipe and 23.2±8.74 μm for the newly formed polystroma; mean±s.d.; Fig. 6A and Fig. S5), but cortical cells in the stipe are about half the diameter than those in the blade (11.43 μm±3.26 μm versus 15.55 μm±4.34 μm, respectively, in the *y*-axis; 12.83 μm±3.58 μm versus 22.02 μm±7.41 μm in the *z*-axis).

At the base of the stipe, the cortex and meristoderm expand slightly (Fig. 9A,B), a sign of nascent haptera, which are blunt, intertwined digitate protrusions that fix algae 20 days old and older to the substratum (Fritsch, 1945; Davies et al., 1973). Sagittal sections show that, at this stage, the embryo fixes itself directly to the substrate at the bottom of the cortex (Fig. 9A,B).

### In the TZ, the meristoderm cells differentiate centripetally into medullary cells in only three cell divisions

**Fig. 10.**
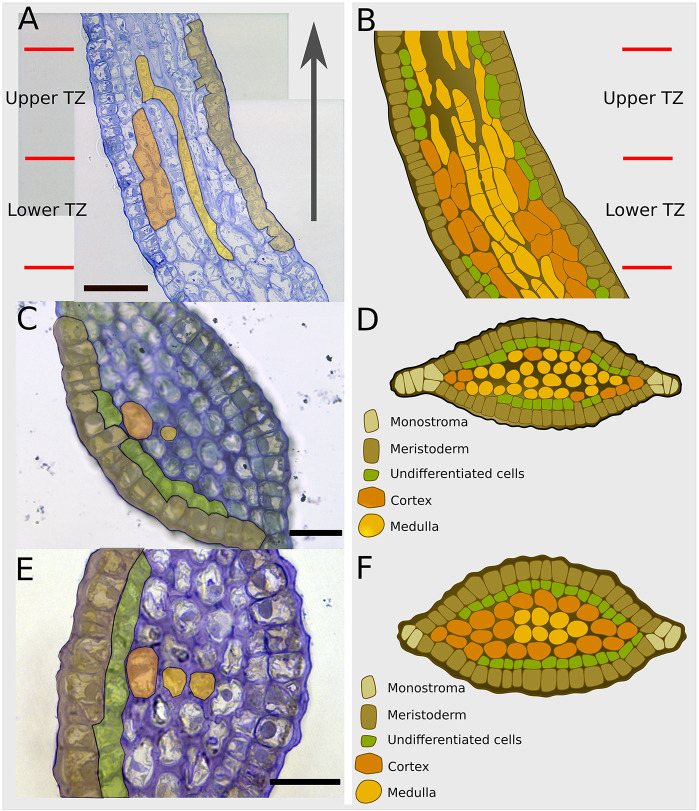
**Semi-thin longitudinal and transverse sections in Phase III transition zone (TZ).** (A) Longitudinal section of a 20-day-old embryo, divided into an upper TZ and a lower TZ. The grey arrow indicates the base-to-apex direction. (C,E) Transverse sections of the upper (C) and lower (E) TZ. (B,D,F) Colour-coded schematics of the TZ corresponding to the sections in A,C,E, respectively. Note the undifferentiated cells (green), which are more abundant than in the blade and stipe in Figs 8 and 9, respectively. Scale bars: 50 μm (A); 25 μm (C,E). Representative images from two specimens per orientation of section. In A,C,E, cell types are pseudocoloured as a semi-transparent overlay.

Longitudinal and transverse sections show that the TZ displays a specific spatial organisation with differences in the upper and lower regions. Whereas the upper TZ, which is under the base of the blade, has few cortical cells, wide intercellular spaces and many medullary cells (Fig. 10A-D), the lower TZ located right above the stipe is filled with tightly packed cortical and few medullary cells (Fig. 10A,B,E,F). This difference illustrates a bipolar gradient of the TZ, with blade-like features (i.e. high abundance of medulla) in the upper part and stipe-like features (i.e. abundant cortical cells) in the lower part. This gradient suggests that the TZ plays a role in the formation of these two distinct tissues, perhaps through different dynamics*.*

Another feature of the TZ is that both the upper and lower TZ contain a high proportion of as-yet UCs that result from the asymmetrical periclinal division of meristoderm cells (Figs 6A,B, 7C). Interestingly, the TZ and the stipe show a relatively balanced ratio of periclinal to anticlinal divisions, whereas in the blade, almost two anticlinal divisions occur for every periclinal division (Fig. S7). This might result from the higher abundance of UCs in the TZ (Fig. 7C) and definitively explains why TZ and stipe are cylindrical organs whereas the blade is flat. However, the reason why the TZ has such a high proportion of UCs remains unknown. In the upper TZ, UCs may participate in the formation of the medulla by differentiating directly into medulla cells and skipping the cortical cell stage (Fig. 7C, dotted red line). Their undifferentiated status might also be a pre-requisite to allow the TZ to carry out high apico-basal growth activity.

**Fig. 11.**
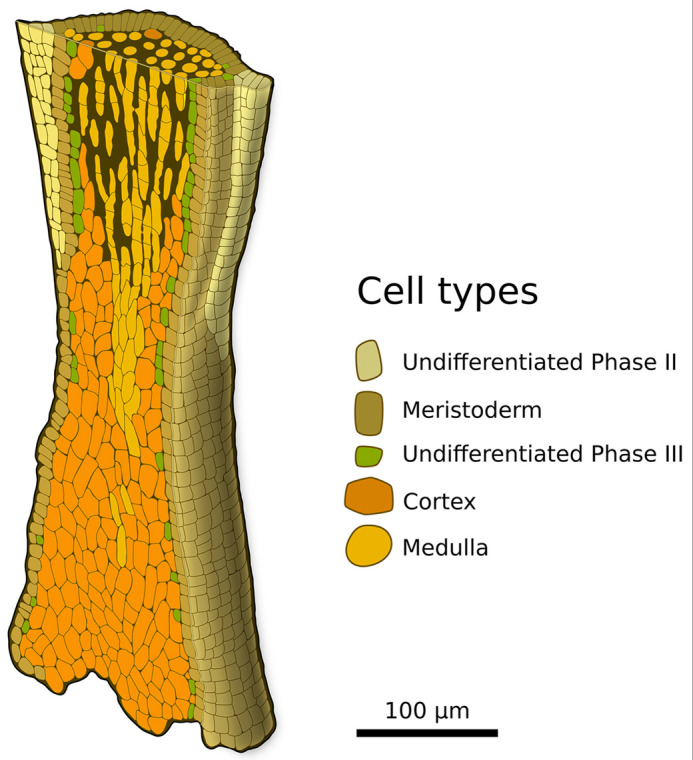
**Partial 3D representation of the transition zone (TZ).** In the lower part of the TZ, the peripheral meristoderm cells mainly differentiate into cortical cells only; in the upper part they differentiate into medullary cells.

Fig. 11 shows the overall organisation of TZ in 3D. The gradual change in cellular content is apparent with medulla being more abundant in the upper TZ and cortex in the lower TZ and stipe. Together with Figs 3 and 7, this drawing illustrates the high level of regulation that must operate to control tissue growth activity and tissue differentiation on a spatial scale as low as 100 µm in all three spatial axes. However, and interestingly, the time scale of growth and tissue differentiation is low, because several days are necessary to achieve 3D growth and even more to develop differentiated tissues (Fig. S2 shows the small increase in tissue thickness in steps of 10 days).

### Blade cells share their contents through plasmodesmata

**Fig. 12.**
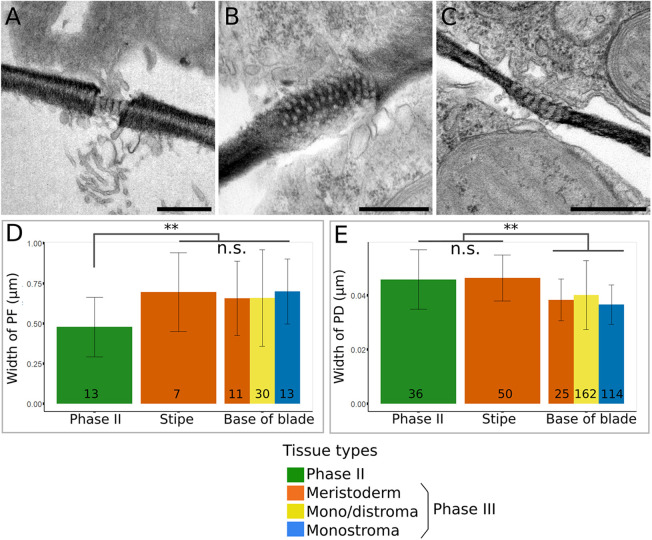
**Width of plasmodesmata and pit fields.** (A-C) TEM acquisitions showing pit fields (PFs) crossing the cell wall of two neighbouring cells: (A) PF between cells of a Phase II embryo; frontal section (*xy*), (B) PF between cells of a Phase III monostroma (transverse section, *yz*), (C) PF between meristoderm cells of the stipe (transverse section, *yz*). (D,E) Average width and standard deviation of PF and plasmodesmata (PD) in Phase II monostromatic tissue, and in different cell types of Phase III tissues taken at the base of the blade (i.e. monostroma, distroma and meristoderm). (D) Width of PFs. PFs in Phase II tissues are significantly narrower (42%) than in Phase III tissues at the base of the blade. There is no significant difference between the means of stipe and base of blade. (E) Width of PD. PD are 18.64% wider in Phase II tissues than those from Phase III tissues at the base of the blade. There is no significant difference between the means of stipe and Phase II. The number of measurements are shown at the base of each bar. Two specimens were measured for each region. **P<0.01 (Welch's *t*-test). n.s., not significant (*P*>0.05). Scale bars: 0.5 μm. The TEM images come from ultra-thin sections from two specimens.

Cell communication is a crucial trait in the control of early development. In cell-walled organisms, the most efficient way to communicate is through plasmodesmata, which are cytoplasmic connections that span the cell walls of two neighbouring cells. Transmission electron microscopy (TEM) observations of transverse sections of *Saccharina* Phase II and III embryos show the presence of plasmodesmata concentrated in pit fields along our axial system (Fig. 12A-C). The widths of pit fields and plasmodesmata were measured for different tissues and regions of the blade. We measured the width of pit fields on sections that were perpendicular to the interface area between two cells to avoid bias in measurements. It was extremely difficult to obtain perfectly aligned sections, but we managed to make a selection according to the sharpness and the shape of pit fields and plasmodesmata. Our measurements showed that, whereas pit fields increase in width as the blade grows from Phase II to Phase III (Fig. 12D, Table S3; mean 0.48±0.19 μm versus 0.68±0.26 μm, respectively), the width of the plasmodesmata decreases (Fig. 12E, Table S4; mean 45.88±10.94 nm versus 38.67±10.69 nm). Therefore, the higher number of plasmodesmata may compensate for their narrower diameter, resulting in overall wider pit fields. Wider pit fields suggest increased symplasmic trafficking and potentially cell-to-cell communication as the embryo develops. Whether these are necessary for the complex control of cell growth and cell differentiation in the three spatial dimensions is still unknown, but this result strongly suggests that material can diffuse from one cell to its neighbours.

## DISCUSSION

The work presented above provides a comprehensive description of the first steps of cell differentiation in the whole embryo of the brown alga *Saccharina latissima* (overall schematics shown in Fig. S8).

### Tuning cell division and cell differentiation differently

*Saccharina* is an interesting system in which to study the formation of a 3D body, because it establishes 3D growth only after a monolayered blade is formed approximately 15 days post-fertilisation. Furthermore, 3D growth is accompanied by complex and rapid cell differentiation (relative to cell growth), while the overall embryo morphology remains unchanged up to the juvenile stage.

Little is known about the development of brown algae or kelps; most of our knowledge on tissue structure and emergence is based on studies dating back to the 1950s or earlier. The embryo is initially monostromatic and during its development turns into a huge, complex parenchymatous thallus. Using classic histological approaches, we focused on the multiplication and differentiation of tissue layers in the later stages of embryogenesis of *Saccharina*. Polystromatisation, although not unique to brown algae, is still a distinctive process owing to the late introduction of a third growth axis.

In an oversimplified and general way, cells have two fates: either they divide or they differentiate. We have not been able to measure the mitotic activity of the meristoderm cells present in the TZ, but we noticed the presence of UCs, which are the direct product of division of the meristoderm layer. We were nonetheless able to monitor the differentiation of these cells. We observed that meristoderm cells differentiate very quickly relative to the cell division rate. Our approaches prevent us from measuring the absolute time necessary for meristoderm cells to differentiate into medullary cells, which represent the terminal differentiation state in vegetative kelps. However, in relative terms and in early embryos, it takes a maximum of three distinctive cell layers.

Another remarkable fact of *Saccharina* is that additional undifferentiated cell layers come from the mitotic activity of a single, peripheral layer, the meristoderm (our observations; Smith, 1939; Fritsch, 1945). Growth in only peripheral cells is common to other brown algae, such as the Fucales *Fucus* and *Sargassum*, and the Dictyotales *Dictyota dichotoma*, in which only one or two apical cells ensure all growth in all three axes (Katsaros and Galatis, 1985; Bogaert et al., 2020). In contrast, the shoot or root apical meristems of land plants are usually made up of several cell layers (Steeves and Sussex, 1989). We estimate that the meristoderm is a synapomorphic tissue among parenchymatous brown algae with a key role in complementing the main apical meristems in organogenesis and girth growth. Nevertheless, apical meristems are absent in kelps, so the meristoderm that initiates in the TZ and spreads acropetally in the blade and basipetally in the stipe is the only tissue responsible for the growth of all new tissues.

Thus, what distinguishes the 3D growth of *Saccharina* from 3D growth of other brown algae (Fucales, Dictyotales) is the high rate of cell differentiation, regardless of the position of cells within the algal body. Is this high rate of cell differentiation due to the implementation of 3D growth taking place well after the beginning of development, i.e. when the embryo has grown as an undifferentiated cell monolayer after several rounds of cell division? Whereas most plant organisms maintain ‘pockets’ of undifferentiated cells while the rest of their cells commit to a given fate very early in embryogenesis, *Saccharina*, in which cells remain uncommitted for a long period of time (i.e. embryogenetic Phases I and II), appears to combine the high dynamics of cell proliferation in the meristoderm with the high dynamics of cell differentiation of its daughter cells. In plants, the cambium is a mitotically active secondary meristem that differentiates rapidly into secondary vascular tissues (Steeves and Sussex, 1989). However, the cambium has crucial differences with the meristoderm. First, it functions in two directions, yielding cells of different types (secondary xylem and phloem) by alternating periclinal cell divisions in the inner and outer directions. Second, it emerges after primary growth has developed all the organs of the adult organism, and its role is limited to the maturation of existing tissues/functions.

### What triggers polystromatisation?

In *Saccharina*, the transition to 3D growth occurs approximately 14 days after the first cell division of the zygote, when the embryo contains at least 1000 cells. What triggers this transition?

3D growth is considered a complex morphogenetic process. Most metazoans and land plants have developed complex 3D structures, but some organisms have not. Among the latter, some develop simple 3D stem-like structures (mosses, e.g. *Physcomitrium*; Moody, 2019), bilayer cell sheets (e.g. the chlorophyte green alga *Ulva*; Wichard et al., 2015) or loose spheres (e.g. *Volvox*; Umen, 2020). Although these organisms have most of the genetic tools necessary to control cell division spatially (Nishiyama et al., 2018; Szövényi et al., 2019), they do not. Interestingly, when some unicellular organisms, both prokaryotic and eukaryotic, build multicellular architectures when selected for, they favour 3D organisation to the detriment of other, apparently geometrically more simple, structures (Márquez-Zacarías et al., 2021; Bozdag et al., 2023). Thus, the persistence of the 2D monolayer cellular sheet in the *Saccharina* embryo, like all the apparently simple body organisations observed in mosses or green algae, may provide evidence of strict control of cell division planes, and of the existence of a negative control on 3D growth.

Interestingly, Sauvageau (Fritsch, 1945) reported that in the kelp species *Saccorhiza bulbosa* (currently *S. polyschides*, Tilopteridales; Bringloe et al., 2020), the first periclinal cell divisions occur near the rhizoids located in the most basal cells of the embryo. Similarly, the TZ of the ribbon-like thallus of *Chorda* (Chordales, ex-Chordaceae in order Laminariales) is established at the base of the monostromatic tissue adjacent to the rhizoids, and it gradually grows distally as polystromatisation proceeds (Kogame and Kawai, 1996). Hence, the differentiation of rhizoids and their increased number may be the signal that triggers the initiation of polystromatisation in kelps. In *Saccharina* (Laminariales), once initiated, polystromatisation progresses acropetally as a wave starting from the rhizoid end (base of the embryo, future holdfast) to the apex end. Furthermore, several brown algae have been shown to produce a signal inhibiting branching and tissue regeneration through basipetal diffusion (Katsaros, 1995; Tanaka et al., 2017). However, all display active apical growth (apical meristem) in contrast to kelps. Nevertheless, a polystromatisation-inhibiting signal may be produced in the apex of the *Saccharina* embryo, so that the distance between the apex and the basal part of the embryo increases while the lamina keeps growing. This spatialisation would result in a decrease of the concentration of the apical inhibitory signal that allows 3D growth to take place in the basal part. Alternatively, a polystromatisation wave could be induced by a signal diffusing acropetally, as does the sporogenesis inhibitor in kelps (Buchholz and Lüning, 1999; Pang and Lüning, 2004), which is potentially auxin (Kai et al., 2006). In both cases, this type of wave mechanism requires a system of signal transport.

We showed that, as the embryo develops, the width of the pit fields increases. This may be due to formation of *de novo* plasmodesmata being added to existing pit fields (Nagasato et al., 2015), or to new, larger pit fields being positioned as the new cell wall forms during cell divisions. Regardless of the cause, an increase in the size of pit-field area is a sign of an increase in tissue complexity (Terauchi et al., 2015). Different sizes of molecules can travel through plasmodesmata, as observed in *Fucus* (Fucales) (Nagasato et al., 2015) and *Sphacelaria* (Sphacelariales) (Nagasato et al., 2017), and the transport of metabolic molecules, such as fixed carbon and phosphate organic compounds, may also occur via diffusion through plasmodesmata to reach the TZ where new growth takes place (Lüning et al., 1973). In the undifferentiated cells of the monostroma, signalling molecules and the supply of metabolic compounds may induce a shift in the orientation of cell division plane, thus allowing growth into the third spatial dimension.

### Conclusion

Here, we describe a unique case of third-axis growth for cell-walled organisms. Polystromatisation is a distinctive phenomenon through which *Saccharina* sets up 3D growth, and establishes a meristoderm, a peripheral meristem, which differs from apical meristems mainly by its high rate of cell differentiation. The superficial meristem of *Saccharina* is not unique. It is a common tissue found in other parenchymatous brown algae that compartmentalise the function of their apical meristems. However, the meristoderm is the main tissue through which growth can occur in three spatial axes in kelps. Hence, the meristoderm is an evolutionary innovation that plays a key role in the plethora of parenchymatous brown algal body plans. Our work sheds new light on our understanding of how kelps set up 3D growth and generates questions regarding its induction and control at the molecular and cellular levels.

## MATERIALS AND METHODS

### Algal cultures

Kelps are conspicuous macroscopic brown algae, mainly belonging to the order Laminariales. The life cycle of this clade is dominated by the sporophyte phase, and the male and female gametophytes are microscopic filaments (i.e. heteromorphic dioecous life cycle; Fig. 1C). *Saccharina*, like all kelps, has a heteromorphic diplobiontic cycle (Fig. 1C) (Theodorou and Charrier, 2021).

One mature sporophyte was collected from the beach at Perharidy (Roscoff, Brittany, France) (48°43′33.5″N 4°00′16.7″W), and spores were isolated and treated as described by Theodorou et al. (2021). After fertilisation of the female gametophytes with male gametes, embryos were cultivated in Petri dishes in full Provasoli-enriched, sterile, filtered seawater for up to 30 days. Because the wild sporophyte is heterozygous, the embryos have different genetic backgrounds and are considered biological replicates.

### Semi-thin sections

Live embryos were observed for up to 14 days of development under a bright-field microscope. For histogenesis observations, 20-day-old embryos were fixed with 1% glutaraldehyde (Sigma-Aldrich) and 1% paraformaldehyde (Sigma-Aldrich) in sterile, 0.2 μm-filtered seawater for 2 h at 13°C. Then, the fixation medium was gradually changed to 0.1 M sodium cacodylate. Post-fixation consisted of incubating the thalli in 1% OsO_4_ in 0.1 M sodium cacodylate at 4°C overnight. After washing with 0.1 M sodium cacodylate, the fixed embryos were dehydrated using an ethanol: sodium cacodylate gradient. For the infiltration step, Spurr resin gradually replaced ethanol (Spurr, 1969), with fresh resin for the last step before polymerisation. Semi-thin sections of 500-750 nm were mounted on glass slides and stained with 1% w/v Toluidine Blue in a 1% w/v borax (sodium tetraborate) water solution or Stevenel's Blue (del Cerro et al., 1980), and then observed under a light microscope (Leica DMI6000B inverted optical microscope) equipped with a motorised stage and a DFC450C colour camera, controlled by LASX version 3.0 software.

To image the emergence of 3D growth, transverse, sagittal and frontal semi-thin sections were prepared from five different embryos (three, two and one specimens for each type of section, respectively). From them, up to five sections were prepared for the morphometric analyses with FIJI (Schindelin et al. 2012; Table S1). Each section on the same specimen and region was considered a technical replicate. Each sectioning orientation was prepared from at least two embryo specimens. Distance was over 50 µm between transverse sections and about 26 μm between sagittal sections. To avoid measuring the same cells, we selected what seemed to be median sections (smallest cell diameters). In all cases, very misshapen or folded specimens were not chosen for embedding or sectioning. For imaging, the white balance of acquired images was adjusted post-acquisition using a macro that balances RGB colours based on a selected region. The macro is available on GitHub (Patrice Mascalchi; https://github.com/pmascalchi/ImageJ_Auto-white-balance-correction). Contrast and brightness were then manually optimised for better visualisation of tissue and cellular structures. Figs 2I, 4B, 8A and 10A are the result of stitching several acquisitions. The acquisitions come from the same specimen but different regions and are then combined on Inkscape. The original acquisitions are available through our deposited raw data (https://doi.org/10.6084/m9.figshare.24212541.v1). The *z*-projected images in Fig. 2D,E,H were generated through stack focuser on FIJI (Schindelin et al. 2012).

### TEM protocol

For TEM, ultra-thin sections of 50-70 nm thickness from the embedded material described above were mounted on copper grids (Formvar 400 mesh; Electron Microscopy Science©) and stained with 2% uranyl acetate for 10 min (Woods and Stirling, 2013) and 2% lead citrate for 3 min at room temperature (Reynolds, 1963). Sections were observed using a JEM-1400 Flash TEM microscope (JEOL Ltd.).

### Statistical analysis

For cell morphometrics, we used the Shapiro and Wilk (1965) method and QQ plots to estimate the normality of our measurements (Table S2A-C). Because the distribution was not normal, we used the non-parametric test of Kruskal and Wallis (1952) to detect statistically significant differences between the arithmetic means of the different groups of tissue and positions. Multiple pairwise comparisons of means for different positions of the same tissue type were carried out using Dunn's test (Dunn, 1961) (Table S2C). The Holm–Bonferroni (Holm, 1979) method was used for adjusting the *P*-value. For plasmodesmata, Welch's *t*-test (Welch, 1947) was used for pairwise comparison of means. The statistical analysis took place through R version 4.2.1 on R-studio.

### Drawings

Illustrations of the sections were drawn using Biolographix (biolographix.blogspot.com) with Inkscape software version 1.2 (Inkscape Project) and a KAMVAS Pro 13 GT-133 pen tablet (©Huion).

## Supplementary Material

10.1242/develop.201519_sup1 Supplementary information

Table S2. Measurement of the cell dimensions in the X-, Y- and Z-axes from semi-thin sections of *Saccharina* embryos and details on the statistics.

Table S3. Measurements of the width of pit fields (diameter in 2D) from TEM pictures of *Saccharina* embryos

Table S4. Measurements of the width (diameter in 2D) of plasmodesmata from TEM pictures of Saccharina.
