# Supplementary material for: The shift to 3D growth during embryogenesis of kelp species, atlas of cell division and differentiation of Saccharina latissima
Source: Development. 2023 Oct 26;150(21):dev201519. doi: 10.1242/dev.201519 (PMC10660787; doi:10.1242/dev.201519)
Supplement: Supplementary information [file develop-150-201519-s1.pdf]

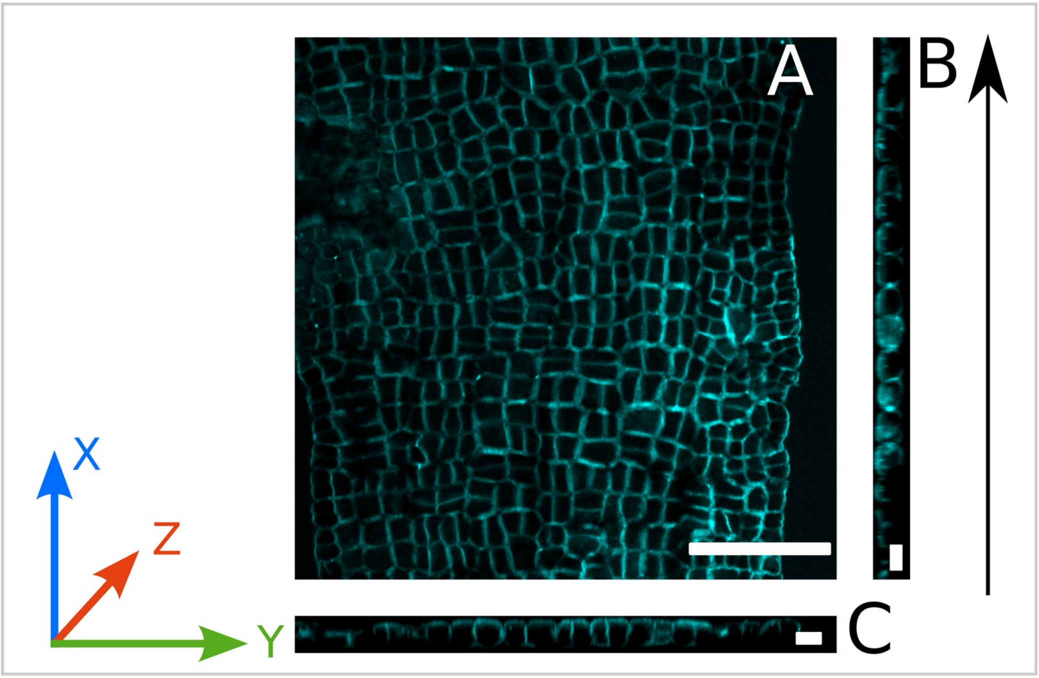

Fig. S1. Morphology of the monolayer cell sheet as observed under confocal microscopy after a 45 min incubation with 20 μM calcofluor white solution (fluorescent brightener 28, F-3543, Sigma-Aldrich; excitation wavelength: 380 nm, emission wavelength: 475 nm), washed three times in seawater for 15 min at room temperature and observed using confocal microscopy (SP5 confocal microscope, Leica Microsystems; laser 405 nm, PMT emission passing band 675-745 nm). A) Frontal (X-Y) section of an embryo fixed in 1.5% PFA. B and C) Orthogonal views of this section. B) Longitudinal (X-Z) section, C) Transverse (Y-Z) section. The black arrow indicates the growth direction of the apico-basal axis. Bars: A: 90 μm; B and C: 16 μm.

Development • Supplementary information

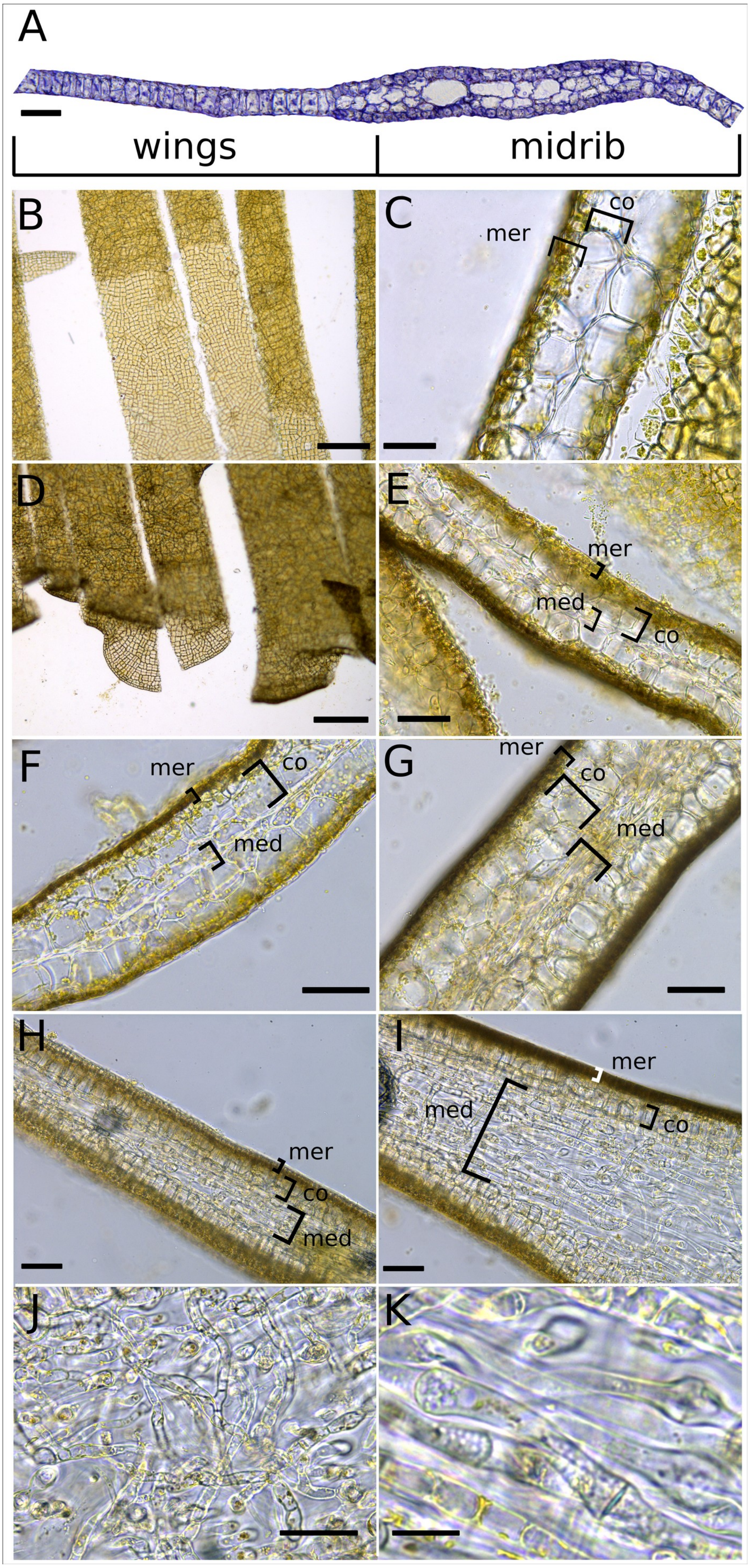
A
wings
midrib
B
C
co
mer
D
E
mer
med
co
F
mer
co
med
G
mer
co
med
H
mer
co
med
I
mer
med
co
J
K

Fig. S2. Cell differentiation and histogenesis atthe blade: from the four-celled layers to thicker, differentiated layers. The following sections come from the region with the largest width (Y-axis). Live material of different ages (days after fertilisation) was sectioned transversely using a scalpel in seawater. A) Semi-thin transverse section from young polystroma of 20-day-old embryos. The wings of the blade are mostly monostromatic while the midrib is polystromatic with a ~ four-celled layer. B and C) 30 days after zygote polarisation (azp). B) Bright field (BF) acquisition on the surface of transversely sectioned fresh blade in seawater. The lighter areas are monostromatic and distromatic and darker areas are the expanding polystroma. C) BF image from the side of the section of the polystroma shown in (B). D and E) 50 days azp. D) Same as (B). The polystroma has considerably expanded and only a small distromatic area remains. E) The side of the section at the polystroma in (D). Five tissue layers are distinguishable. The outer and darker tissue present on both sides is the meristoderm (mer), which is at least one cell layer thick. The inner tissue is a one-cell-layer thick cortex (co) and the medulla (med) with filamentous cells. F and G) 60 days azp. F) Fresh transverse section from the side of the blade. Co consists of at least two cell layers and med is barely visible. G) Fresh transverse section at the midrib. Co consists of several cell layers and the medulla is conspicuous. The meristoderm contains at least two or three cell layers. H-K) Four-month-old lab-cultivated specimen. H) Fresh transverse section from one of the "wings" or sides at the base of the blade. At least one cell layer of meristoderm is visible and several layers of cortex. I) Section from the midrib. Medulla appears extremely expanded due to its flexible nature and the preparation of the sample for the microscope. The cortex consists of about two cell layers and is still less prominent than the medulla. J) Detail from the meshwork of medulla from another transverse section at the midrib. K) Cropped and zoomed detail from I. These are representative images from two specimens and two sections per region. A comes from manually stitched images of the same section. Brackets indicate the different tissues. mer: meristoderm, co: cortex, med: medulla. Images were acquired under a BF microscope. The contrast and brightness of the BF images were modified. Bars: A: 40 µm; B, D: 300 µm; C:45 µm; F, E, G: 70 µm; H, I, J: 70 µm; K: 23 µm.

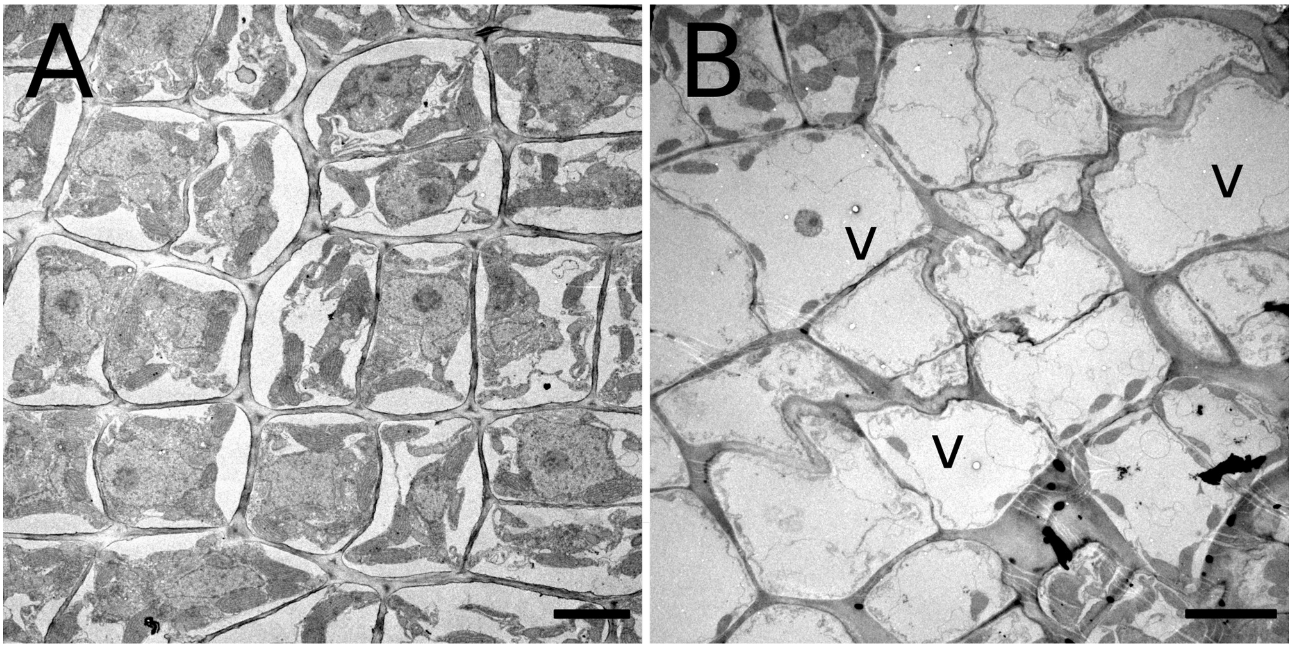

Fig. S3. Transmission electronic microscopy observations on the general morphology of meristoderm and cortex tissues. A) Frontal section of meristoderm tissue. The meristoderm cells are cuboid with a dense cytoplasm rich in chloroplasts. Retraction of the protoplast is visible in some cells, due to the fixative solution. B) Frontal section of cortical cells, depicting large transparent cells. Large vacuoles are annotated with a ‘V’. A few chloroplasts can be observed at the periphery of the cells. Additional features of these two cell types are given in Table 2. Bars: A: 5 µm; B: 10 µm.

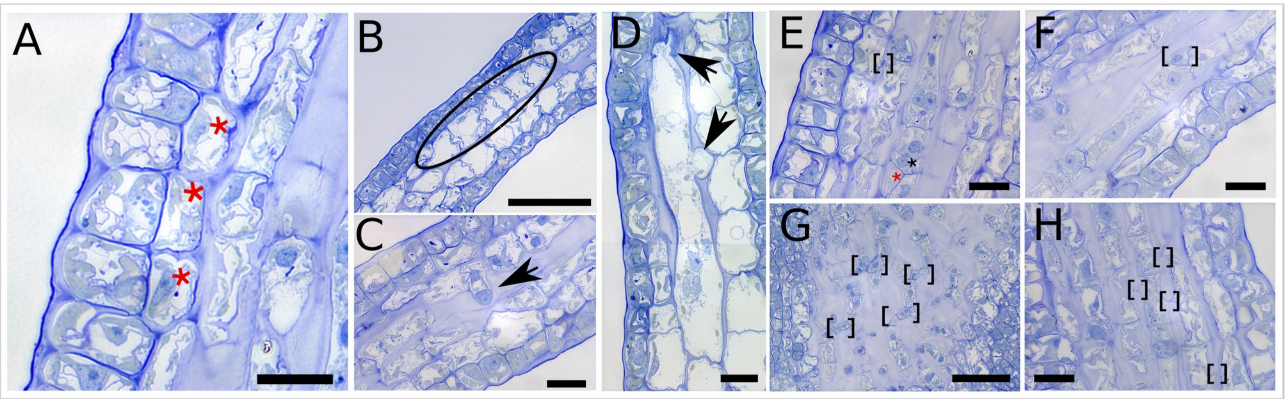

Fig. S4. Longitudinal sagittal sections of the blade. A) Divisions of the meristoderm. The shape of the meristodermal cells suggests that these cells undergo many transverse anticlinal divisions. Red stars illustrate asymmetrical periclinal cell divisions producing the undifferentiated cells. B) Section from the boundary between mature and newly formed polystromata. The cortical cells (C) in the black circle seem to have undergone a series of transverse anticlinal divisions that contribute to growth in the X-axis. Note the thick cell wall between the two longitudinal layers of C cells, which does not support self-renewal of the cortical tissue by periclinal cell division of C cells. C, D) Pre-medullary cells with protrusions and characteristics of cortical cells (arrows). In D, the large elongated cell appears to be multinucleate. In the bottom left-hand corner, note the progressive differentiation of UC cells into C cells (from top to bottom in the photo). These 4 cells are aligned with the meristodermal cells, confirming that the UC and C cells are derived from meristodermal cells. E-H) Traits of medulla. E) Growing medullary elements. A transversal connection (pit field) between a cortical cell and a medullary element is shown within brackets (black star: anticlinal division, red star: periclinal division). F) Initials of branching medullary cells (brackets: cell division of a branch). G) Frontal section of branching medulla at the base of the blade. Different branches are shown within brackets. H) Horizontal connections (pit fields) between medullary elements. Bars: A, G: 50 µm; B,C,D,E,F,H: 14 µm.

Development • Supplementary information

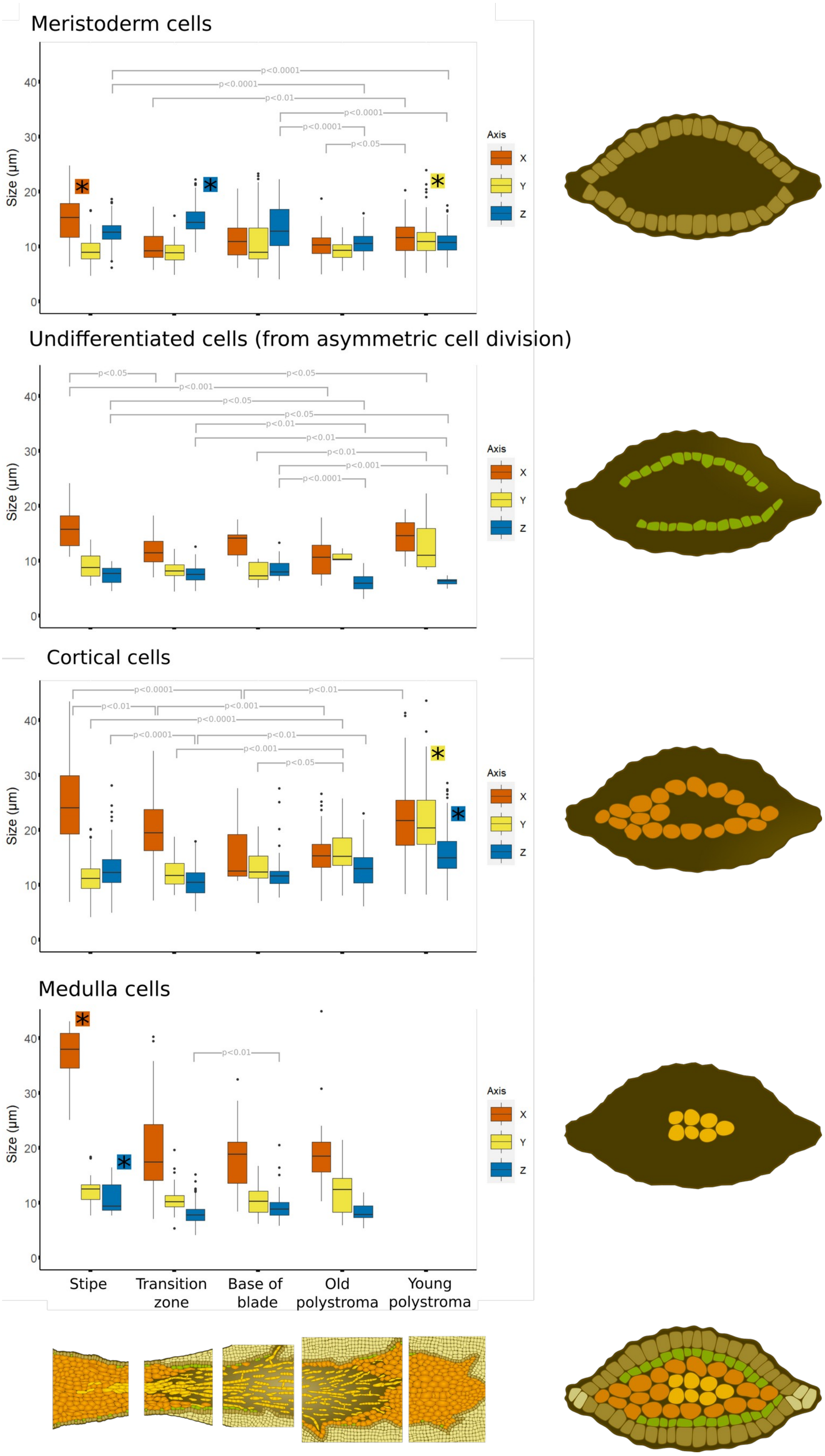

Development • Supplementary information

Fig. S5. Morphometrics of the different cell types in each region.

Dimensions (μm) of the different cells (meristoderm, undifferentiated from asymmetric cell division, cortex and medulla) measured in the X-, Y- and Z-orientations from semi-thin sections of four embryos 20 days after zygote polarisation (Table S2), used to draw the cells shown in Fig. 6. The box encloses the 25th and 75th percentiles of the measurements surrounding the arithmetic median (dark horizontal line). A non-parametric Dunn's (1961) test was used for the comparison of means. Asterisks indicates significant differences (adjusted p-value <0.05, Holm-Bonferroni (Holm, 1979) adjustment method). Brackets indicate individual pairwise mean comparisons and the customary p< 0.05 or p<0.001, etc. The analyses were carried out in R (vers. 4.2.1; (R Core Team, 2017)), using the rstatix package (vers. 0.7.0; (Kassambara, 2023)) and the tidyverse (vers. 1.3.2; (Wickham et al., 2019)). At least three length measurements from two sections on at least two specimens are presented.

Development • Supplementary information

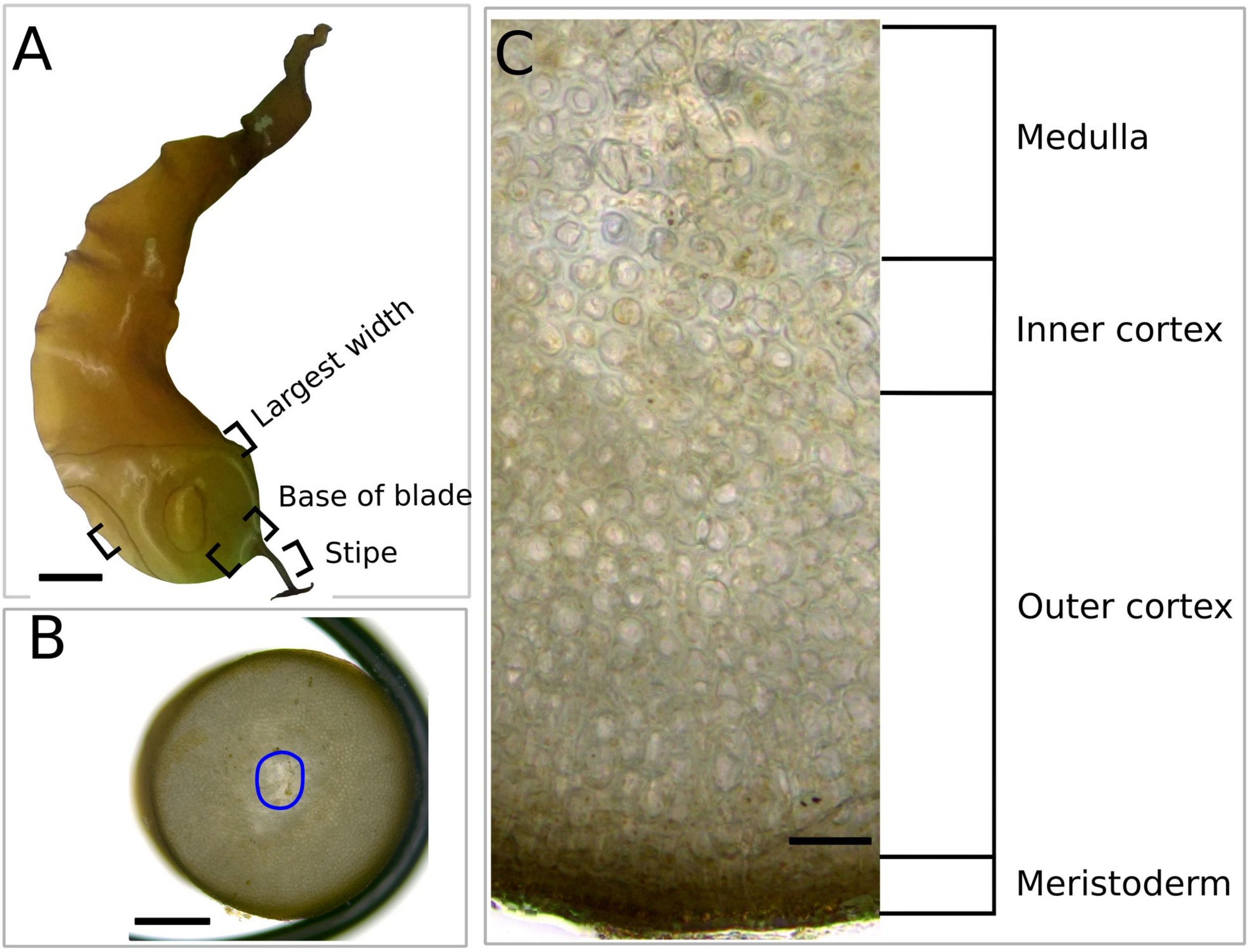

Fig. S6. Stipe of a four-month-old specimen cultivated in the lab. Sections were prepared as described in Fig. S2. A) Sectioned specimen. B) Transverse section of fresh material (not fixed) showing the centre of the stipe. The circle indicates the centre of the stipe where medullary tissue resides. C) Zoom of B). The different layers are indicated. All images and sections come from the specimen in A. Image in C) was produced using the Fiji plugin "stack focuser" (https://imagej.nih.gov/ij/plugins/stack-focuser.html) from a bright field image stack. Bars: A: 40 µm; B: 8 mm; C: 300 µm.

Development • Supplementary information

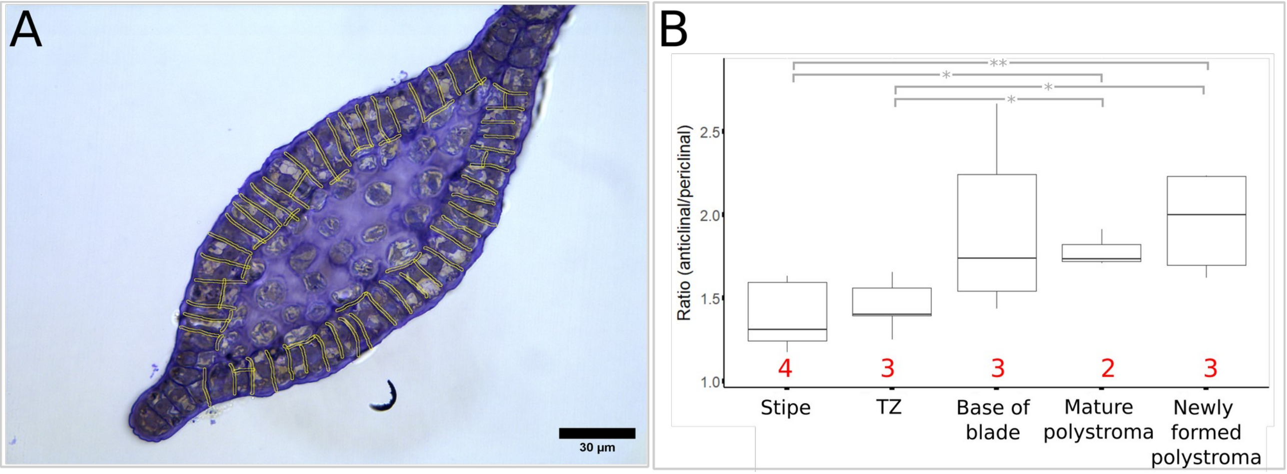

Fig. S7. Ratio of periclinal and anticlinal cell walls between the meristoderm and the innermost tissues in the blade. A) Representative example of how periclinal and anticlinal cell walls were counted. B) Box-plot of the ratio of anticlinal to periclinal cell walls counted in the different tissues. Note that the polystromata of the blade have no statistically significant difference between them. Red letters indicate the number of specimen, from which up to 6 sections of varying orientation were observed *: p-value< 0.05, **: p-value< 0.01, Student t-test, no adjustment of the p-value.

Development • Supplementary information

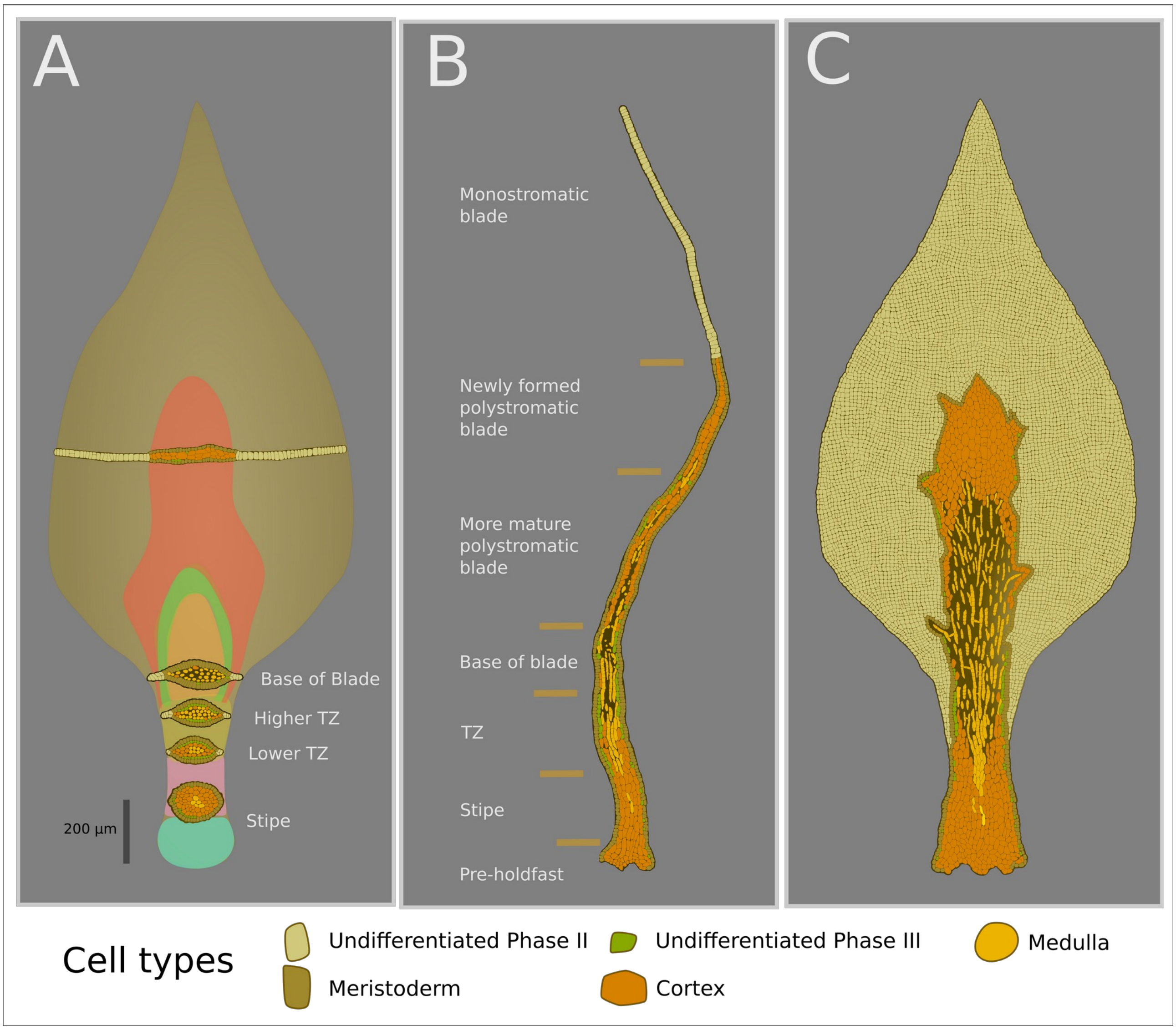

Fig. S8. General view of the organisation of the tissues in a *Saccharina latissima* embryo. Drawings summarising the tissue distribution and organisation in the early embryo of *Saccharina latissima*, from A) transverse sections (positioned over a frontal shadow), B) longitudinal sagittal sections, C) longitudinal frontal sections.

Supplementary references

Holm, S. (1979). A Simple Sequentially Rejective Multiple Test Procedure. *Scandinavian Journal of Statistics* **6**, 65–70.

Kassambara, A. (2023). rstatix: Pipe-Friendly Framework for Basic Statistical Tests. R package version 0.7.2.

R Core Team (2017). *R: A Language and Environment for Statistical Computing*. Vienna, Austria: R Foundation for Statistical Computing.

Wickham, H., Averick, M., Bryan, J., Chang, W., McGowan, L. D., François, R., Grolemund, G., Hayes, A., Henry, L., Hester, J., et al. (2019). Welcome to the Tidyverse. *Journal of Open Source Software* **4**, 1686.

Development • Supplementary information

Table S1. Number of specimens and sections for each spatial tissue orientation. S: sagittal; T: transverse; F: frontal

		Specimen #			Section #		
	**Section orientation**	**S**	**T**	**F**	**S**	**T**	**F**
**Tissue**	Stipe	2	3		5	3	
	TZ	2	2		5	3	
	Base	2	2	1	5	4	1
	Basal, mature polystroma	2	1		4	1	
	Newly formed polystroma	2	2		4	3	

Table S2. Measurement of the cell dimensions in the X-, Y- and Z-axes from semi-thin sections of *Saccharina* embryos and details on the statistics.

Dimensions of the cell in the X-, Y- and Z-axes (when available from sections) were measured using Fiji (ImageJ). Schematic presentation of an average size cell for each type and region is presented in Fig. 6. Graphical representation of the results and their statistical analysis are presented in Fig. S5. (.xls).

A) Measurement of the cell dimensions in the X, Y and Z axes from semi-thin sections of Saccharina embryos. B)Summary statistics of cell morphometrics. Means presented are arithmetic means. SD: standard deviation. X: longitudinal axis parallel to the longest axis of the tissue, Y: Perpendicular to X , Z: perpendicular to XY. C) Table S2C: Multiple pairwise comparisons of the means for different positions on the developing embryo based on the same tissue type using Dunn's (1961) test. Shapiro-Wilk tests and QQplots indicate non-normal distribution (results not shown). We chose the Kruskall-Wallis test (1952), which indicates that for the different groups of tissue and position, there are statistically significant differences between the arithmetic means of the different groups (results not shown). The results of these comparisons are plotted in Supplementary Figure S5. The Holm-Bonferrroni (1979) method was used for adjusting the p-value (p.adj).

Available for download at
https://journals.biologists.com/dev/article-lookup/doi/10.1242/dev.201519#supplementary-data

Development • Supplementary information

Table S3. Measurements of the width of pit fields (diameter in 2D) from TEM pictures of *Saccharina* embryos

The width of pit fields was measured using Fiji (ImageJ) on TEM images of sections of different tissues and developmental stages. The mean and SD are indicated in Fig. 12.

Available for download at
https://journals.biologists.com/dev/article-lookup/doi/10.1242/dev.201519#supplementary-data

Table S4. Measurements of the width (diameter in 2D) of plasmodesmata from TEM pictures of *Saccharina*.

Means are arithmetic means. SD: standard deviation. X: longitudinal axis parallel to the longest axis of the tissue, Y: perpendicular to X, Z: perpendicular to XY.

Available for download at
https://journals.biologists.com/dev/article-lookup/doi/10.1242/dev.201519#supplementary-data
