# Supplementary material for: The shift to 3D growth during embryogenesis of kelp species, atlas of cell division and differentiation of Saccharina latissima
Source: Development. 2023 Oct 26;150(21):dev201519. doi: 10.1242/dev.201519 (PMC10660787; doi:10.1242/dev.201519)
Supplement: Supplementary file 1 [file develop-150-201519_review_history.pdf]

The shift to 3D growth during embryogenesis of kelp species, atlas of cell division and differentiation of *Saccharina latissima*

Ioannis Theodorou and Bénédicte Charrier
DOI: 10.1242/dev.201519

Editor: Ykä Helariutta

Review timeline
Original submission: 8 December 2022
Editorial decision: 21 February 2023
First revision received: 14 June 2023
Editorial decision: 17 July 2023
Second revision received: 16 August 2023
Accepted: 12 September 2023

Original submission

First decision letter

MS ID#: DEVELOP/2022/201519

MS TITLE: Outside in: how kelp embryos shift to 3D growth, an atlas of cell division and differentiation

AUTHORS: Ioannis Theodorou and Benedicte Charrier

I have now received all the referees' reports on the above manuscript, and have reached a decision. The referees' comments are appended below, or you can access them online: please go to BenchPressand click on the 'Manuscripts with Decisions' queue in the Author Area.

As you will see, the referees express considerable interest in your work, but have some significant criticisms and recommend a substantial revision of your manuscript before we can consider publication. If you are able to revise the manuscript along the lines suggested, which may involve further experiments, I will be happy receive a revised version of the manuscript. Your revised paper will be re-reviewed by one or more of the original referees, and acceptance of your manuscript will depend on your addressing satisfactorily the reviewers' major concerns. Please also note that Development will normally permit only one round of major revision. If it would be helpful, you are welcome to contact us to discuss your revision in greater detail. Please send us a point-by-point response indicating your plans for addressing the referee’s comments, and we will look over this and provide further guidance.

Please attend to all of the reviewers' comments and ensure that you clearly highlight all changes made in the revised manuscript. Please avoid using 'Tracked changes' in Word files as these are lost in PDF conversion. I should be grateful if you would also provide a point-by-point response detailing how you have dealt with the points raised by the reviewers in the 'Response to Reviewers' box. If you do not agree with any of their criticisms or suggestions please explain clearly why this is so.

© 2023. Published by The Company of Biologists under the terms of the Creative Commons Attribution License (https://creativecommons.org/licenses/by/4.0/).

Reviewer 1

Advance summary and potential significance to field

The authors provide nice illustrations of development of Saccharina and the differentiation of the conductive tissue (medula with trumpet hyphae). The reviewer is very confused about the content of the paper, most likely it should be reworked and better explained. But I can only interpret from my current understanding of the text.

The work is very timely as the organism has been poorly described and the species has a economic value and under increasing interest outside of Asia.

We are asked to review with 2 questions in our head:

Firstly, what is the advance made in the paper and how significant is this for the field?

Some studies from the beginning of 20 th century have described development. However the the transition between different stages has not been described in detail.

The text is written confusingly. If I have understood things well there are 2 possibilities:
1. The growth is mediated via a "meristoderm" that divides and which daughter cells differentiate into undifferentiated cell --> cortex -> medula. The authors also suggest (line 396) the medullary cell might actually differentiate "ultrafast" from meristoderm cells.

Abstract: " The study of the progression through this sequence shows how 3D growth propagates through a series of asymmetric cell divisions from a peripheral monolayer of cells, the meristoderm (external epidermal meristem). Cells descending from asymmetric cell division in actively growing regions rapidly differentiate into cortical and medullary cells."

2. There is no true meristoderm and the whole region is meristematic. The cortex divides mitotically, the medula divides mitotically and the outer "cortex" (here called 'meristoderm') divides as well in the transition zone.

The authors hypothesise the solution 1 (402) in most of the text. "Another remarkable fact of kelp is that all thickening (in the Y-Z plane) comes from the mitotic activity of a single, peripheral cell layer, the meristoderm."

But also do not exclude solution 2 (line 307)
"This observation suggests that the differentiation of these cells into cortical cells and then into medullary cells (as observed in Fig. 8C,E) is slower in the TZ than in the blade and in the stipe, thereby providing the TZ with the functional traits of a meristematic tissue. Alternatively, the higher abundance of these undifferentiated cells in the TZ may be due to a higher level of cell division in a periclinal orientation. "Line 256: "On average, medullary cells in the blade are filamentous and are thinner than cortical cells, but of similar length (along the X-axis) (Fig. 5 and Suppl. Fig. 2; Fig. 6H-I, Suppl. Fig. 4C,G,H). They seem to expand primarily along a single axis, usually parallel to the embryo apico-basal axis (Fig. 6H). They generally divide anticlinally, but periclinal divisions also occur, probably as a function of branching (Suppl. Fig. 4E,F, red star,G)" So medula filaments divide. (line 268) " Therefore,we hypothesise that transverse divisions, cell elongation and shape changes lead to the differentiation of the cortex into the medulla." So they hypothesise they divide from cortex.

Figure 4: cortex cells divide just like the meristoderm cells.

In line 307the authors explain the two possilities expliciteely "a high proportion of as yet undifferentiated cells that result from the asymmetrical periclinal cell division of meristoderm cells. This observation suggests that the differentiation of these cells into cortical cells and then into medullary cells (as observed in Fig. 8C,E) is slower in the TZ than in the blade and in the stipe, thereby providing the TZ with the functional traits of a meristematic tissue. Alternatively, the higher abundance of these undifferentiated cells in the TZ may be due to a higher level of cell division in a periclinal orientation. This difference in division plane is reminiscent of Smith's

© 2023. Published by The Company of Biologists under the terms of the Creative Commons Attribution License (https://creativecommons.org/licenses/by/4.0/).

observation on mature kelp, whereby the cell division rate is higher at the base of the blade and in the TZ than in any other region (Smith 1939)" and quite rightly add that "It is difficult to discriminate between these two hypotheses, because the observation of sections does not provide an assessment of the rate of differentiation of undifferentiated cells into cortical cells or, in turn, of cortical cells into medullary cells."

Therefore sadly, I cannot conclude the authors have explained their significant advance. They explain a controversy. 2 possibilities and cannot discriminate between both. Although solutions 1 (meristoderm hypothesis) is presented as the case in this organism.

Imo; The idea that meristoderm cells as the source of new cells divide a tiny cell, which expands into a cortex cell and than would get smaller and elongated and arrange itself in filaments allowing a function as conductive tissue is far fetched. Undoubtedly all cell types here divide in the meristematic region. Probably the 2nd layer of "asymmetric cells" is a extra layer of outer cortex cells. But perhaps these cells may expand a bit and differentiate into cortex; leaving a bit of interpretation that there would be some "meristoderm" activity as well.

Comments for the author

A second question reviewer are asked: do the data reported in the paper justify the conclusions drawn?

I would say not. But am very confused about what conclusions have been drawn (see above). Conclusions are drawn (~"differentiation from meristoderm") and than contradicted again (~"everything divides after all in the transition zone"). As if the paper was written by 2 people with opposing interpretation trying to come to some carefully negotiated solution.

To be able to draw conclusions one would have to execute time lapse observations instead of providing only 'static' data (sections).

However the authors may better explain their hypothesis and the novelty.

Additional remarks:

-This way of developping is described as novel. However I do not understand why it is novel when it has been described at the beginning of the century and many red algae also develop first monostromatic and then become polystromatic at the base.
-"Brown algae display the exceptional feature of developing 3D body structures long after the onset of embryogenesis". This may be true for Saccharina, but not for Fucus, Sargassum, Dictyota, Dictyopteris,…
Again contradiction of the above.
- The interpretation of the Katsoros paper is wrong: 2 meristems are mentioned (apical and meristoderm). However there are 3: also marginal apical initials (Katsaros & GAlatis on the matter: Three types of "meristems" function: (a) the central apical initials, which give rise to the other meristems and to initial cells of the midrib; (b) the marginal apical initials, which contribute mainly to wing formation; and (c) a superficial meristem which is a true meristoderm and contributes to midrib formation.)
- Dictyota is mentiioned to have a meristoderm (line 404) vs Dictyota has no meristoderm (line 529)
- Figure 4: the above and below cells do not divide. The are better removed it looks funny by the last drawing.
- I did not found mention of how many individuals were observed.
- "Polystromatisation is not a uniform process. Figure 3B shows that, on a 20-day-old embryonic blade, monostromatic tissue can interrupt tissues that are already engaged in polystromatisation." But what if the embryo was slightly bent? Then you would have cut through the polystromatic region twice and it would look interrupted.
- The chosen method is very weird:
A confocal light microscope is used to observe sections stained with calcofluor.
But they are stained with OsO_4 in cacodylate buffer (very toxic, only usefull for TEM). The TEM is only used to show there are vacuoles in medula and lots of chloroplasts in cortex, but that is a very

© 2023. Published by The Company of Biologists under the terms of the Creative Commons Attribution License (https://creativecommons.org/licenses/by/4.0/).

basic statement. It feels very weird to read the authors handled these toxic slides under de confocal for no obvious reason. Why not cryotome, or technovit embedding. Or better: why no time lapses (at least for the early stages).

Reviewer 2

Advance summary and potential significance to field

This paper describes the developmental steps towards 3D growth in a kelp - more specifically an understudied representative of the brown algae. The sheer volume of work that has gone into this manuscript is extremely impressive - this is an exciting advance in the field, and will be impactful. 3D growth (although defined in a somewhat murky way) is a land plant innovation - yet brown algae also evolved 3D growth convergently. Previous studies have looked at species such as Sargassum muticum, which produces a well defined tetrahedral apical cell - a hallmark of 3D growth in some lineages of land plants. This study in a kelp offers a novel perspective on the issue - some clarification is required though. When is the switch from 2D to 3D?

Comments for the author

The authors present a beautiful developmental study here, and the level of detail is quite impressive, and the content is highly novel and exciting. Some suggestions are made below to improve the overall clarity of the manuscript. Generally, it would be nice if the authors could truly define 3D growth - the transition from 2D to 3D (for example) is relatively unclear, and needs to be spelt out more directly.

GENERAL/INTRODUCTION:

1. 3D growth is an invariable hallmark trait of all land plants, and there is a wealth of pre-existing literature discussing how this is achieved (not only in angiosperms but also in others such as bryophytes: the representative group of extant land plants). Since kelps are not from the green lineage (ancestors of land plants), it must be emphasized how this is just another variant of 3D growth observed in an independent lineage (a case of convergent evolution).

2. One of the consequences of 3D growth and essentially why it was selected for during the terrestrialization of plants is that it allowed them to occupy aboveground niches i.e., growing upright without the support of water. Kelps, on the other hand, are still flat and unable to support themselves mechanically without water buoyancy. Would the term “pseudo-3D growth” be more appropriate? 3D growth has already been predefined to be underpinned by the rotation of cell division planes in meristems (i.e., 3D apical growth) giving rise to initials while maintaining cell/cell population identity. In this case, “3D growth” is essentially established by mere thickening/stacking of cell layers. It is not that the authors do not make valid claims – this work represents a really beautiful developmental study – but can they really define 3D growth in this context please.

3. There is a lack of discussion how 3D growth is established in other plant lineages that are non-angiosperms. The diversity of meristematic cell number/activity/dynamics would highlight the similarities, as well as uniqueness of the meristoderm as an analogous structure to the apical meristems.

MATERIALS AND METHODS:

1. Line 120: Their – anthromorphizing. Just use “it” or “The life cycle of this clade…”

2. It was not elaborated how cell sampling for morphometrics were done e.g., how tissues were imaged or how many cells from each region were sampled.

RESULTS:

© 2023. Published by The Company of Biologists under the terms of the Creative Commons Attribution License (https://creativecommons.org/licenses/by/4.0/).

1. Line 168: It is curious to know if there is a regimental number of cells that is required in Phase I before the embryo enters Phase II. Furthermore, after the first unequal cell division, is there a difference in the activity of the generated apical and basal cell based on cell division frequency (in generating the column of cells)?

2. Line 170: Since it was mentioned later in the text that polystromatization (division in the Z-plane) proceeds acropetally, do the first longitudinal anticlinal cell divisions (entry to Phase II) also behave acropetally? Basipetally? Or simultaneously?

Figure 2: It is suggested for this figure to be presented in a landscape orientation (see Figure 4) to convey “progression” of form from 1D to 3D Table 1, Table 2, and Figure 2C, 2G, 2J: The orientation of X- and Y-axes is a bit confusing. Conventionally the surface of the growth medium is used as a reference for the X-axis. Furthermore, kelps grow underwater in an “upright” position (the term “apico-basal” was also mentioned a couple of times) which for the layman will mean the Y-axis.

Figure 2J: There should also be a late-stage diagram where the meristoderm (the actively dividing tissue) is labelled to better visualize the growth points relative to the orientation of the cell division planes).

3. Line 256: There is no graph (with statistical tests) comparing morphometric measurements between cell types. There needs to be a visualized quantitation of these metrics aside from scaled diagrams which is usually based on just averages (i.e., means).

4. Line 263: Wrong figure citation (?) – might be Suppl. Fig. 4 instead of Suppl. Fig. 2H.

Figure 7: “Longitudinal and transversal…” – Might be worth clarifying that it is “longitudinal frontal.”

5. Line 375: It is curious to determine how much connections do each cell have at Phase II.

DISCUSSION:

1. Line 406: Not all land plant apical meristems consist of several cell layers. In mosses, there are single cell meristems (simplex meristems) – as is the case in the remaining bryophytes (liverworts and hornworts), some lycophytes and most certainly monilophytes. Please rephrase accordingly.

2. Line 419: “seemingly making up for lost time.” Since the meristoderm is analogous to the population of cells in the apical meristem, it can be argued that this are just multiple apical cells that is dispersed on the superficial layer of the growing sporophyte that actively divides and is responsible for the growth in multiple axes.

3. Line 434: Don’t use “basal” or “early divergent lineages” to refer to bryophytes. See McDaniel 2021 (10.1111/nph.17241). Also, refrain from anthropomorphization i.e., “struggle”

Supplemental Figure 4E, 4F, 4G, 4H: These structures are not clear/obvious in the image. Brackets is suggested to be used instead of arrows. Diagrammatic versions of these sections might help in visualizing pit fields and branches.

First revision

Author response to reviewers' comments

S ID#: DEVELOP/2022/201519
MS TITLE: Outside in: how kelp embryos shift to 3D growth, an atlas of cell division and differentiation

© 2023. Published by The Company of Biologists under the terms of the Creative Commons Attribution License (https://creativecommons.org/licenses/by/4.0/).

AUTHORS: Ioannis Theodorou and Benedicte Charrier

Answer to reviewers

Black: reviewers
Blue: our answers

Reviewer 1 (named below "Rev 1")

Advance Summary and Potential Significance to Field:

The authors provide nice illustrations of development of Saccharina and the differentiation of the conductive tissue (medula with trumpet hyphae). The reviewer is very confused about the content of the paper, most likely it should be reworked and better explained. But I can only interpret from my current understanding of the text.
Answer to Rev 1. We took notice of your overall impression of our MS, and considered it very seriously. This shows that it is difficult to clearly describe growth in three spatial dimensions. We have added new figures and reorganised the text to be more precise in our description of tissue organisation. We now present our work in 3 sections, based on the chronology of the events: 1. initiation of 3D growth (Z-axis). The first cell division implied in this step is equal and symmetrical. 2. Acropetal (X-axis) and centrifugal (Y-axis) propagation of 3D growth. 3. Maintenance (also named "reinforcement" or "consolidation") of 3D growth (Z-axis). In this step, meristodermic cells divide asymmetrically, from which cortex and medulla differentiate. We are confident that this revised organisation of the manuscript will improve significantly the understanding and the reach of our article.

The work is very timely as the organism has been poorly described and the species has a economic value and under increasing interest outside of Asia.

We are asked to review with 2 questions in our head:

Firstly, what is the advance made in the paper and how significant is this for the field?

Some studies from the beginning of 20 th century have described development. However the the transition between different stages has not been described in detail.

Answer to Rev 1. We understand here that Rev 1 has acknowledged the fact that the aim of our article is to describe the acquisition of 3D growth and the formation of 3D tissue step by step in a novel organism. This involves bringing morphometric and dynamic information about transitions in cell shape, cell size, and (a)symmetry and (un)equality of cell divisions. Here however, we can only propose an atlas, as all the data are extracted from fixed tissues (some sections from fresh, non fixed, material have been added to the revised version). Obtaining data from living tissues is difficult because 1. As in most brown algae, growth is slow: at the embryogenetic stage, cell division takes place ~ every 12-24 hours. 2. Algal cells require a low temperature (~ 13°C) to grow, and 3. Algal cells are full of pigments. Putting these features together, imaging cell division in living brown algae, moreover in 3D tissues with several cell layers, is currently a major technical challenge. We are currently initiating a project in which light-sheet microscopy and bi-photon confocal microscopy will be used to indeed image brown algal tissue in compatible growth (temperature, light) and optical conditions (maintenance of focus) for several days, in order to be able to capture the 2D-3D transitions (which happens ~ 15 days after egg fertilisation, what our work here allows to describe). We hope that such experiment, while more robust and technically developed, will confirm the data presented in the current MS. We think that providing an atlas beforehand will allow to guide future studies in this field.

The text is written confusingly. **If I have understood things well there are 2 possibilities:**
1. The growth is mediated via a "meristoderm" that divides and which daughter cells differentiate into undifferentiated cell --> cortex -> medula.
Answer to Rev 1: That's correct. This refers to the 3rd step into which we now describe polystromatisation in Saccharina. This is where 3D growth is maintained and reinforced along the Z-axis. We revised the Abstract: (lines 19-23) " … growth shifts from 2D to 3D, show that 3D growth

© 2023. Published by The Company of Biologists under the terms of the Creative Commons Attribution License (https://creativecommons.org/licenses/by/4.0/).

first initiates from symmetrical cell division in the monolayered lamina, and then is enhanced through a series of asymmetrical cell divisions in a peripheral monolayer of cells called the meristoderm. Then, daughter cells rapidly differentiate into cortical and medullary cells...”. We further describe this step in other part of the revised version.

The authors also suggest (line 396) the medullary cell might actually differentiate “ultrafast” from meristoderm cells.
Answer to Rev 1: That is a short-cut and is not quite correct. Actually, from their position as observed in the semi-thin sections, we inferred that meristoderm cells first divide. Then, one of the daughter cell, that we named ‘undifferentiated cells” (UC), differentiate into cortical cells, which themselves differentiate into medulla cells. In the lamina, very little cortex is observed, and therefore, we hypothesize that this is due to a direct transition from UC cells to medulla cells, without lingering on the "cortical" stage. This is now clearly explained in Fig. 6B and 6C of the revised version.

2. **There is no true meristoderm and the whole region is meristematic.** The cortex divides mitotically, the medula divides mitotically and the outer “cortex” (here called ‘meristoderm’) divides as well in the transition zone.
Answer to Rev 1: That is correct to some extent only. Indeed, it all depends on which orientation we are looking at it. Our observations combined with the work of Fritsch and Smith led us to propose that in the TZ, all cells divide along the X and the Y axis. However, in the Z axis, mainly the meristoderm cells divide. They are the layer that ensures most of the growth in Z (thickening). That’s why we call it a meristodermn and this is the aim of our paper here. Therefore, in summary, during the maintenance and reinforcement of polystromatisation (step 3 of our revised version) the meristoderm supplies most of the interior of the organism with cells resulting from asymmetric periclinal divisions (UC cells). The daughter cells (UC cells) then differentiate into cortical cells and medulla cells, and can have their own cell growth mainly in the X axis (cell expansion and division; (Fig. 6C sagittal view) but also to a lower extent in the Y and Z orientation (Fig 6C, transverse view). This is noteworthy that this growth activity is much more reducedr than that of the meristoderm. Therefore, we consider that the meristoderm ensures most of the thickening.
We have added a new figure summarising the different growth activity in the 3 spatial direction, along the embryo. From this figure, it can be seen that the growth activity varies along the embryo, for each of the 3 directions. The combinations of the 3 makes the overall embryo shape.

The authors hypothesise the solution 1 (402) in most of the text. “Another remarkable fact of kelp is that all thickening (in the Y-Z plane) comes from the mitotic activity of a single, peripheral cell layer, the meristoderm.”

But also do not exclude solution 2 (line 307)
“This observation suggests that the differentiation of these cells into cortical cells and then into medullary cells (as observed in Fig. 8C,E) is slower in the TZ than in the blade and in the stipe, thereby providing the TZ with the functional traits of a meristematic tissue. Alternatively, the higher abundance of these undifferentiated cells in the TZ may be due to a higher level of cell division in a periclinal orientation. “
Answer to Rev 1: We confirm our sayings, as lines 402 and 307 are not incompatible.
Regarding more specifically the TZ (compared to blade and stipe), when viewed in the Z-axis, cell differentiation is slower because we see many undifferentiated cells that are the result of unequal and asymmetrical cell division of the peripheral cell layer. Therefore, because the TZ has more undifferentiated cells, the TZ has features reminiscent of a meristem, as defined in plants (i.e. slow division rate of usually small, and undifferentiated cells). However, after that cell layer, cell differentiation is rapid (in relative time calibrated on the cell division rate), and therefore, this feature distinguishes the meristoderm from a meristem (i.e. their is no reservoir of undifferentiated cells). In the revised version, we have added a new Fig. S7 that shows that cells in the TZ divides more periclinally than anticlinally, when compared to the basal part of the blade. In addition to promoting a cylindrical shape, this property also supports a higher abundance of undifferentiated cells in the TZ, and hence, of its “meristematic” nature.

Line 256: “On average, medullary cells in the blade are filamentous and are thinner than cortical cells, but of similar length (along the X-axis) (Fig. 5 and Suppl. Fig. 2; Fig. 6H-I, Suppl. Fig. 4C,G,H). They seem to expand primarily along a single axis, usually parallel to the embryo apico-

© 2023. Published by The Company of Biologists under the terms of the Creative Commons Attribution License (https://creativecommons.org/licenses/by/4.0/).

basal axis (Fig. 6H). They generally divide anticlinally, but periclinal divisions also occur, probably as a function of branching (Suppl. Fig. 4E,F, red star,G)" So medula filaments divide. (line 268) " Therefore, we hypothesise that transverse divisions, cell elongation and shape changes lead to the differentiation of the cortex into the medulla."
→ So they hypothesise they divide from cortex.
Answer to Rev 1: We don't explicitly say that medulla divides from the cortex. Instead, we think and say that medulla cells differentiate after the cortical cell divides. Then, what is cited above by Rev1, is the further growing activity of amedulla cell, after it differentiated : i.e. 1) it makes branches to fill the central space of the tissue, 2) it divides transversely so that it multiplies longitudinally, in the same time as 3) it elongates further. This is now illustrated in Fig. 6C, frontal view. Only transverse division is observed, except for the branching where the orientation of cell division might be oblique (combination of X and Y/Z axes).
In summary, the sequence of events is 1) meristodermic cells divide asymmetrically to give one meristodermic cell and one undifferentiated cells, 2) undifferentiated cells expand in size and differentiate to the transparent, rich in vacuoles, cortical cells. These cells can divide anticlinally (Fig. 6C, transverse view) but most of the time they divide transversely (Fig. 6C, sagittal view), 3) cortical cells and their vertical neighbours, go through a gradual elongation and overall shape change as they differentiate to medulla cells (Fig. 6C). 4) medulla cells can divide transversely to accompany the elongation of the organism along the X-axis (Fig. 6C, frontal view), and longitudinally, this potentially being the first step of branching.
We recognise that the sentence " Therefore, we hypothesise that transverse divisions, cell elongation and shape changes lead to the differentiation of the cortex into the medulla." was not precise enough, as this was not clear which of cortical cells or medulla cells divide." We changed the text of the revised version accordingly (many places).

Figure 4: cortex cells divide just like the meristoderm cells.
Answer to Rev 1: Figure 4 has been completely revised. It is now Fig. 5 and we hope that from it, and the revised text, it is now clear that cortical cells do not divide as meristoderm cells.

In line 307, the authors explain the two possilities explicitely "a high proportion of as yet undifferentiated cells that result from the asymmetrical periclinal cell division of meristoderm cells. This observation suggests that the differentiation of these cells into cortical cells and then into medullary cells (as observed in Fig. 8C,E) is slower in the TZ than in the blade and in the stipe, thereby providing the TZ with the functional traits of a meristematic tissue. Alternatively, the higher abundance of these undifferentiated cells in the TZ may be due to a higher level of cell division in a periclinal orientation. This difference in division plane is reminiscent of Smith's observation on mature kelp, whereby the cell division rate is higher at the base of the blade and in the TZ than in any other region (Smith 1939)"
Answer to Rev 1: We have refined our understanding of the combination of the data available in the old literature and of our own observations. In our case, we focuson growth along the Z-axis, since it is in the context of the differentiation meristoderm- cortex ->medulla. On the other hand, Smith tried to connect the increased number of meristoderm layers present at TZ and base of blade of mature specimen with observed intercalary growth by previous researcher in this particular region In addition, it refers to mature kelp, while we focus on embryos only. Therefore, we have removed this sentence, which contributes to the confusion.

and quite rightly add that
"It is difficult to discriminate between these two hypotheses, because the observation of sections does not provide an assessment of the rate of differentiation of undifferentiated cells into cortical cells or, in turn, of cortical cells into medullary cells."
Answer to Rev 1: We have modified our interpretation of our data: the presence of undifferentiated cells must be due to a slower differentiation rate, because a higher cell division rate will lead to a wider, thicker tissue at the level of the TZ, which is not the case. Therefore, in the TZ, the daughter cells of the meristoderm cells remain undifferentiated longer than in the other zones of the embryo.
We thank Rev 1 for raising this point and we have modified the text accordingly.

Therefore sadly, I cannot conclude the authors have explained their significant advance. They explain a controversy. 2 possibilities and cannot discriminate between both. Although solutions 1 (meristoderm hypothesis) is presented as the case in this organism.

© 2023. Published by The Company of Biologists under the terms of the Creative Commons Attribution License (https://creativecommons.org/licenses/by/4.0/).

Answer to Rev 1: There is no controversy. We think that the previous version of the MS was not clear enough to allow an easy and clear picture of the formation of 3D tissue. In the revised version, we now first describe polystromatisation in 3 steps: 1) Initiation (Figs. 3A, 4, 5, 7A); 2) Propagation (Fig. 3B, 4, 5, 7B) and 3) Maintenance / reinforcement / Thickening (Fig. 3B, 6, 7B,C, 8, 9, 10). Figures are specifically referred to each of these steps. Secondly, we clarified in which spatial direction each cell division or cell differentiation process took place with the new figures 3 and 7.
In summary, in Z axis, cell division activities are mainly restricted to the outer cell layer. In the transition zone specifically, the meristoderm is particularly active along the X axis, actually as the other cell layers (cortex, medulla) but to a higher extent as the cells are smaller. This led our predecessors to name the TZ a meristematic region. Our paper aims to reconcile the two views by ordering them in the three spatial dimensions with the help of clear drawings. We hope that the new version of our MS will convince Rev 1.

Imo *(in my opinion)*; The idea that meristoderm cells as the source of new cells divide a tiny cell, which expands into a cortex cell and than would get smaller and elongated and arrange itself in filaments allowing a function as conductive tissue is far fetched. **Undoubtedly all cell types here divide in the meristematic region.**
Answer to Rev 1: We understand Rev 1 doubts. However, our observations do not support her/his hypothesis. Until it is possible to follow cell division in living organisms and in depth, we think that it will be very difficult to describe in more details, and overall, dynamically, how Saccharina grows in depth.

Probably the 2nd layer of "asymmetric cells" is a **extra layer of outer cortex** cells. **But perhaps these cells may expand a bit and differentiate into cortex; leaving a bit of interpretation that there would be some "meristoderm" activity as well.**
Answer to Rev 1: Our results do not support that the 2nd layer is also meristoderm, because we did not see this layer divide. Instead, these "asymmetrical cells" eventually enlarge and become cortical cells. What Rev 1 describes is observed in more mature tissues, when the monolayer of each cell type usually thickens by the overimposition of additional cell layers of similar cell types. We have added Fig. S4, which shows clearly (pannel A and E particularly) the presence of a layer of undifferentiated cells that results from the division of the meristoderm, and that progressively differentiate into cortical cells. This is particularly clear in Fig. S4D, bottom part, where some UC cells are already differentiated in cortical cells. We hope that this supplementary figure will help Reviewer 1 and readers to accept our theory.

Reviewer 1 Comments for the Author:
A second question reviewer are asked: do the data reported in the paper justify the conclusions drawn?

I would say not. But am very confused about what conclusions have been drawn (see above). Conclusions are drawn (~"differentiation from meristoderm") and than contradicted again (~"everything divides after all in the transition zone". As if the paper was written by 2 people with opposing interpretation trying to come to some carefully negotiated solution.)
Answer to Rev 1: In our answers above, we now explain why our interpretation looks like coming from two different persons. Actually, it came from two different angles (spatial orientations)! Yet, we wonder if the misunderstanding does not come also from the fact that Rev 1 keeps mixing cell division and cell differentiation, as her/his sentence above could suggest it: "Conclusions are drawn (~"differentiation from meristoderm") and than contradicted again (~"everything divides after all in the transition zone"). We suppose that she/he meant "differentiation from meristoderm, and hence, "no cell division in the other cell layers". For this reason, we now split cell growth and cell differentiation activity in two distinct figures, Fig. 3 and Fig. 7.

To be able to draw conclusions one would have to execute time lapse observations instead of providing only 'static' data (sections).
Answer to Rev 1: We agree with Rev 1. However, brown algae tissue are full of brown pigments that prevents microscopy in depth. Using confocal microscopy, we could only image ~ 10 µm, whereas *Saccharina* cells are ~ 12 µm in depth (Z-axis) (unpublished). Therefore, we cannot image polystromatisation with confocal microscopy. That's why we went for semi-thin sections. Also, cell division in *Saccharina* embryo is slow (i.e. one division every 12-24 hours). Therefore, to monitor

© 2023. Published by The Company of Biologists under the terms of the Creative Commons Attribution License (https://creativecommons.org/licenses/by/4.0/).

growth of the different layers, the algae would have to stay under the microscope for several days. Growth conditions for these organisms are 13°C and slim light. These conditions are not easy to combine. We are currently working on alternative imaging approach, and our preliminary experiments have shown that light-sheet microscopy and bi-photon microscopy can help. We are currently developping these technologies. They will require time before we can provide complementary data.
However the authors may better explain their hypothesis and the novelty.
Answer to Rev 1: We focus on the process of polystromatisation, for which we aimed to provide observations as detailed as possible. This is the first atlas of 3D growth in a brown alga. We believe that it will pave the way to more advanced studies of morphogenesis, cell growth activity and cell growth orientation, all in an original organism and will raise interest in the community of developmental biologists.

Additional remarks:

-This way of developping is described as novel. However I do not understand why it is novel when it has been described at the beginning of the century and many red algae also develop first monostromatic and then become polystromatic at the base.
Answer to Rev 1: It is difficult to understand what Rev 1 means with her/his reference to many red algae also develop first monostromatic and then polystromatic at the base. Are there any specific references?
From a meticulous research on literature, we find that Fritsch and the works he reviewed, are the only detailed account on the early development of polystromatic red macroalgae (details below). There are though various works on the development of monostromatic and distromatic species of Porphyra (https://doi.org/10.1046/j.1529-8817.1998.341082.x) (https://doi.org/10.1139/b70-055) (https://onlinelibrary.wiley.com/doi/abs/10.1111/j.0022-3646.1984.00609.x). The two cell layers in distromatic species have no apparent difference regarding differentiation plus "distromatisation" is a diffused process without specific origin in contrast with Saccharina embryos where polystromatisation begins at the base of the embryo.
Regarding polystromatic species of Red algae, they belong to Delesseriaceae family. In short, from Fritsch and the works he reviewed, we found that the early development of species belonging to the Delesseriaceae family (specifically, Nitophyllum, Delesseria, Phycodrys) (pp. 536-538, check pp. 536, and 538 for specific references) share characteristics with kelps early development. Specifically, their germlings resemble monostromatic blades like those of Saccharina. In contrast to diffused cell growth of Saccharina, there are active apical cells and intercalary divisions both at early stages and mature thalli while the stalk of these species apears polystromatic. However how and when these additional cell layers emerge is unknown.
Since the present information is scarce we cannot safely come to the conclusion that polystromatisation at the two groups (Saccharina-like kelps and Delesseriaceae from red algae) is similar.

-"Brown algae display the exceptional feature of developing 3D body structures long after the onset of embryogenesis". This may be true for Saccharina, but not for Fucus, Sargassum, Dictyota, Dictyopteris,…
Again contradiction of the above.
Answer to Rev 1: Only a few brown algae display this exceptional features among brown algae and kelp are one of them
We should have written "Kelp" instead of "brown algae". We have completely revised this part of the introduction, with details on the different mode of growth in the different brown algae. Please see the introduction and comments below.

- The interpretation of the Katsros paper is wrong: 2 meristems are mentioned (apical and meristoderm). However there are 3: also marginal apical initials (Katsaros & GAlatis on the matter: Three types of "meristems" function: (a) the central apical initials, which give rise to the other meristems and to initial cells of the midrib; **(b) the marginal apical initials, which contribute mainly to wing formation;** and (c) a superficial meristem which is a true meristoderm and contributes to midrib formation.)
Answer to Rev 1: Which paper does Rev 1 refer to? We have cited two Katsaros & Galatis paper (Katsaros, Chr., and Galatis, B. 1985. Ultrastructural studies on thallus development in Dictyota dichotoma (Phaeophyta, Dictyotales). Br. Phycol. J. **20**(3): 263–276.

© 2023. Published by The Company of Biologists under the terms of the Creative Commons Attribution License (https://creativecommons.org/licenses/by/4.0/).

doi:10.1080/00071618500650271. and Katsaros, Chr., and Galatis, B. 1988. Thallus development in Dictyopteris membranacea (Phaeophyta, Dictyotales). Br. Phycol. J. **23**(1): 71–88. doi:10.1080/00071618800650091.). One on Dictyota (1985), in which derivatives of one or two apical cells ensure most of the 3D tissue growth. And one on Dictyopteris (1988), where the apical initials (central and marginal) constitute the apical meristem of Dictyopteris, while the meristoderm is one superficial meristem as described in detail in the main text of our MS. The meristematic function of the apex in Dictyopteris is further compartmentalised by Katsaros and Galatis because it fits their analysis and description for which we don't have a need in our analysis. We kindly suggest Rev 1 to read Katsaros' review "Apical cells of brown algae with particular reference to Sphacelariales, Dictyotales and Fucales" where he refers to the apical meristem of Dictyopteris and Zonaria farlowii from Dictyotales at pp 50. We kept the Dictyopteris example vs Zonaria as better described histologically wise on all three axes and simpler as paradigm. However, we can include it for completion but prefer not to to avoid text overloading.

- Dictyota is mentiioned to have a meristoderm (line 404) vs Dictyota has no meristoderm (line 529)

Answer to Rev 1: We apologise for the confusion. We have now re-written the whole section of the introduction referring to the 3D growth in Dictyotales and Fucales. It is now: "For example, the brown algal order Dictyotales is characterised by a main apical meristem with one or more apical cells (Fig. 1A,B). Two case examples, *Dictyota* and *Dictyopteris* base their apico-basal growth on an apical meristem but they have different strategies regarding lateral growth and thickening. *Dictyota*'s outer and inner cell divisions take place in the apical meristem while *Dictyopteris* relies on the mitotic activity of the meristoderm. Similarly to *Dictyopteris*, Fucales like *Sargassum* have an apical meristem and a meristoderm (Linardic et al. 2019). Interestingly, cell divisions at the apical meristem of *Sargassum* follow a certain pattern similar to *Physcomitrella*, with a tetrahedral continuously dividing apical cell."

- Figure 4: the above and below cells do not divide. The are better removed it looks funny by the last drawing.

Answer to Rev 1: We have revised the figure accordingly (now Fig. 5).

- I did not found mention of how many individuals were observed.

Answer to Rev 1: The amount of individuals studied with semi thin sections, is for obvious reasons much more restricted than it could be from living algae. For the sagittal orientation, many sections had to be done and observed before reaching the polystromatised region. Four individuals embedded in resin have been observed with semi thin sections. This information has been added to the revised MS with an additional table (Table S1) showing the number of embryo specimen and the number of sections for each tissue. We also have revised the Material and method section accordingly : "
To image the emergence of 3D growth, transverse, sagittal and frontal semi-thin sections were prepared from 5 different embryos (3, 1 and 1 specimen for each type of section respectively). From them, up to 4 sections were prepared for the morphometrics analyses (Table S1). Each section on the same specimen and region is considered a technical replicate. Each sectioning orientation was prepared from at least two embryo specimen. Distance was over 50 µm between transverse sections and of about 26 µm between sagittal sections."

- "Polystromatisation is not a uniform process. Figure 3B shows that, on a 20-day-old embryonic blade, monostromatic tissue can interrupt tissues that are already engaged in polystromatisation." But what if the embryo was slightly bent? Then you would have cut through the polystromatic region twice and it would look interrupted.

Answer to Rev 1: With an ultramicrotome we can make adequate small changes to the orientation of the section to take a proper longitudinal section. Indeed the thickness of a section can pose the issue raised by the reviewer. However, that section is 500 nm thick (we prepared semi-thin sections), and cells are about 10 µm in length to each side (more for the cortex), therefore physically it is impossible in that case to have sectioned a bent region. Additionally we can distinguish on a section when a region on the blade is bent because there are heterogeneities to the presented outer cell wall and the shape of the cells. In the Material and method section of our revised version, we have added this sentence: "
To avoid measuring the same cells, we selected what seemed to be median sections (smallest cell diameters). In all cases, very bent or folding specimen were excluded from embedding or

© 2023. Published by The Company of Biologists under the terms of the Creative Commons Attribution License (https://creativecommons.org/licenses/by/4.0/).

sectioning."

- The chosen method is very weird: A confocal light microscope is used to observe sections stained with calcofluor.

But they are stained with OsO4 in cacodylate buffer (very toxic, only usefull for TEM). The TEM is only used to show there are vacuoles in medula and lots of chloroplasts in cortex, but that is a very basic statement. It feels very weird to read the authors handled these toxic slides under de confocal for no obvious reason. Why not cryotome, or technovit embedding. Or better: why no time lapses (at least for the early stages).

Answer to Rev 1: Rev 1 is right that the protocol used for the prepartation of the semi-thin sections includes OsO4 treatment. It is a standard procedure for preparing material embedded in resin. OsO4 is mostly used as postfixation step when TEM is used afterwards. However, it also offers a more stable tissue and is visually better (black color) which helps orientate the material during embedding and polymerisation.

Therefore:

About fixed embryos: we prepared our sections for both TEM and light-microscopy. All were treated with OsO4 and after embedding, we decided which will be used for TEM and which will be stained with toluidine blue. The revised text now make more clear that only one type of tissue preparation was used for both observations.

About living embryos: we stained them with calcofluor (Fig. S1) and observed with confocal microscopy. This does not require tissue fixation and resin embedding. Therefore, Osmium treatment has not been used with confocal microscopy.

The protocol for calcofluor staining and confocal observation is now in the legend of Fig. S1, as this result is not shown in the main text of our MS. We hope that this will lift any confusion about the different treatment and methods of observation.

About time-lapse observation of living tissue with confocal microscopy: Calcofluor is a stain that is excited at 405nm. Most of the light (excitation and emission) is absorbed by the pigments of the chloroplasts, impairing further the observation of several cell layers. In addition, UV light is phototoxic to brown algae (as for many organisms as it is highly energetic). Therefore, imaging living brown algal tissues in depth is currently an issue, that must be lifted by using more advanced microscopy tools. This is in progress.

Reviewer 2 (named below "Rev 2")

Advance Summary and Potential Significance to Field:

This paper describes the developmental steps towards 3D growth in a kelp - more specifically an understudied representative of the brown algae. The sheer volume of work that has gone into this manuscript is extremely impressive - this is an exciting advance in the field, and will be impactful. 3D growth (although defined in a somewhat murky way) is a land plant innovation - yet brown algae also evolved 3D growth convergently. Previous studies have looked at species such as Sargassum muticum, which produces a well defined tetrahedral apical cell - a hallmark of 3D growth in some lineages of land plants. This study in a kelp offers a novel perspective on the issue - some clarification is required though. When is the switch from 2D to 3D?

Answer to Rev 2: We thank Rev 2 to mention the work done in *Sargassum* showing that 3D growth is established like in Physcomitrium, i.e. through successive periclinal cell divisions of an apical tetrahedral cells. We have added this information in the introduction of our revised version. "

Line 76-78: "Similarly to *Dictyopteris*, Fucales like *Sargassum* have an apical meristem and a meristoderm (Linardic et al. 2019). Interestingly, cell divisions at the apical meristem of *Sargassum* follow a certain pattern similar to *Physcomitrella*, with a tetrahedral continuously dividing apical cell."

Reviewer 2 Comments for the Author:

The authors present a beautiful developmental study here, and the level of detail is quite impressive, and the content is highly novel and exciting. Some suggestions are made below to improve the overall clarity of the manuscript. Generally, it would be nice if the authors could truly define 3D growth - the transition from 2D to 3D (for example) is relatively unclear, and needs to be spelt out more directly.

Answer to Rev 2: We are grateful to Rev 2 for his/her many comments and assessments of our work

© 2023. Published by The Company of Biologists under the terms of the Creative Commons Attribution License (https://creativecommons.org/licenses/by/4.0/).

and MS, and to gain in clarity. We will develop the answer to the above question below.

GENERAL/INTRODUCTION:

1. 3D growth is an invariable hallmark trait of all land plants, and there is a wealth of pre-existing literature discussing how this is achieved (not only in angiosperms but also in others such as bryophytes: the representative group of extant land plants). Since kelps are not from the green lineage (ancestors of land plants), it must be emphasized how this is just another variant of 3D growth observed in an independent lineage (a case of convergent evolution).
Answer to Rev 2:
First, in the abstract, we refer to this feature with "a novel mode of 3D growth in an organism phylogenetically distant from plants and animals.
". Second, in the introduction, we have added at the beginning of the paragraph about brown algae (line 57-66):
"Brown algae are a recent multicellular group that evolved independently from animals and plants (Bringloe et al. 2020; Derelle et al. 2016; Baldauf, 2003). Along their evolutionary trajectory, they convergently developed 3D tissues though different mechanisms, implying changes in the orientation of cell division in specific locations of the embryo. Interestingly, different modes of 3Dgrowth in this single clade. For example, growth can be diffuse, where all cells divide and expand, or can be located in very precise positions within the thallus, in tissues reminiscent of the meristems of land plants (Charrier et al. 2012). Also, 3D growth can be established at the onset of embryogenesis as in animals and plants, or later when the embryo is already made of several cells organised in a linear stack or a monolayered lamina. As a result, 3D growth produces thick, multilayered tissues."
We then detail different strategies developed by brown algae to achieve 3D growth.
In all cases, 3D growth is considered here as growth in thickness, and not only positioning of cells in distinct spatial orientations, as this is the case in the leafy shoot of Physcomitrium.

2. One of the consequences of 3D growth and essentially why it was selected for during the terrestrialization of plants is that it allowed them to occupy aboveground niches i.e., growing upright without the support of water. Kelps, on the other hand, are still flat and unable to support themselves mechanically without water buoyancy. Would the term "pseudo-3D growth" be more appropriate?
Answer to Rev 2: we define 3D from a geometrical perspective, where growth takes place in the 3 spatial dimensions so that it results in thick, multilayered tissues. The role of 3D tissues in the holding of the overall body is not considered in our definition. However, some kelp tissues, as the stipe and the holdfast, are able to hold themselves without the support of water, as observed at low tide (nice illustration of protruding kelp fields at low tide can be found easily on internet, see for example
https://duckduckgo.com/?t=ffab&q=kelp+at+low+tide+stipes&iax=images&ia=images&iai=https%3A%2F%2Flive.staticflickr.com%2F2119%2F2140082220_51041b23d8.jpg or https://external-preview.redd.it/b-w5p26p7GF8LARJrfTNptzk6H6tOI3nDFdBqlRZsIk.png?format=pjpg&auto=webp&s=ccae6c55719c53c70169fd2b31ea5c43b422798a).
Therefore, we don't think that "pseudo-3D growth" is appropriate, as it suggests that 3D growth is not complete, while at the geometrical level and to some extent at the functional level (erect posture), it fulfills completely the concept of 3D growth as defined in the other organisms. Furthermore, our aim here is to provide a descriptive study allowing us to perform comparative studies of 3D growth mechanisms between independent clades and having different names for the same process at the cellular level would endanger the impact of the findings.

3D growth has already been predefined to be underpinned by the rotation of cell division planes in meristems (i.e., 3D apical growth) giving rise to initials while maintaining cell/cell population identity. In this case, "3D growth" is essentially established by mere thickening/stacking of cell layers.
Answer to Rev 2: this is also in this way that we consider 3D growth: formation of a tissue in which cells are arranged in the three spatial dimensions.

It is not that the authors do not make valid claims – **this work represents a really beautiful developmental study** – but can they really define 3D growth in this context please.

© 2023. Published by The Company of Biologists under the terms of the Creative Commons Attribution License (https://creativecommons.org/licenses/by/4.0/).

Answer to Rev 2: We hope that our answers above are enough. Please read the corresponding parts of the introduction.

3. There is a lack of discussion how 3D growth is established in other plant lineages that are non-angiosperms. The **diversity of meristematic cell number/activity/dynamics** would highlight the similarities, as well as uniqueness of the meristoderm as an analogous structure to the apical meristems.
Answer to Rev 2: it is not clear whether Reviewer 2 asks for description of 3D growth in non-angiosperms land plants, like the gymnosperms for example, or in other plant organisms from the Archaeplastida kingdom (that includes land plants, green and red algae), or in turn in other brown algae. If brown algae, we have given some examples of different 3D growth modes in the introduction: for Dictyotales, Fucales and Kelp. Additional description would make the introduction heavy and would require a separate review paper. And a review on meristematic apical cells has already been published by Katsaros, Phycological Research 1995 (doi:10.1111/j.1440-1835.1995.tb00004.x).
If in the Archaeplastida kingdom, then we think that this is out of the scope of this paper, and will deserve a full review. We think that our current manuscript will rather be a stone to add to a future review comparing 3D growth strategies in plant organisms (taken here are photosynthetic, cell walled organisms), as it was done in text books about embryogenesis in animals (e.g. Developmental Biology, book by Scott & Gilbert and modelled in Pierre et al., Dev Cell 2016). However, the diversity of 3D growth stategies is much higher in plant organisms.

MATERIALS AND METHODS:

1. Line 120: Their – anthromorphizing. Just use "it" or "The life cycle of this clade…"
Answer to Rev 2: done

2. It was not elaborated how cell sampling for morphometrics were done e.g., how tissues were imaged or how many cells from each region were sampled.
Answer to Rev 2: Four specimen were fixed and embedded in resin and sectioned either as thin (for TEM) or semi-thin (for staining with toluidine blue). In addition, four living specimen (older than 20 days) were sectioned with a scalpel and observed for further understanding of how 3D growth emerges.
To image the emergence of 3D growth, transverse, sagittal and frontal semi-thin sections were prepared from 3, 1 and 1 specimen respectively. To perform morphometrics, we used two sagittal sections (same specimen), a transverse section and a surface section (frontal longitudinal, for the meristoderm tissue) for the base of the blade, two sagittal sections (same specimen) and two transverse sections (from different specimen) for the stipe, two sagittal sections (same specimen) and two transverse sections (same specimen) for younger polystroma, two sagittal sections (same specimen) for older polystroma. To image the maintenance and reinforcement of 3D growth in the TZ, we cut two serial transverse sections of the same specimen (distance between sections was over 50 µm), and two longitudinal sagittal serial sections of the same specimen (separated by a distance of about 26 µm). To avoid measuring the same cells, we kept only what seemed to be median sections (smallest cell diameters).
This information has been added to the revised version of the MS, mainly summarised in Table S1 (new). How the tissues were prepared and imaged is explained in the Material & Method section: (lines 133-141)
"For histogenesis observations, 20-day-old embryos were fixed with 1% glutaraldehyde (Sigma) and 1% paraformaldehyde (Sigma) in sterile, 0.2 µm-filtered seawater for 2 h at 13 °C. Then, the fixation medium was gradually changed to 0.1 M cacodylate-Na. Post-fixation consisted in incubating the thalli in 1% OsO4 in 0.1 M cacodylate-Na at 4 °C overnight. After washing with 0.1 M cacodylate-Na, the fixed embryos were dehydrated using an ethanol:cacodylate-Na gradient. For the infiltration step, Spurr resin gradually replaced ethanol (Spurr 1969), with fresh resin for the last step before polymerisation. Semi-thin sections of 500–750 nm were mounted on glass slides and stained with 1% w/v toluidine blue in a 1% w/v borax (sodium tetraborate) water solution or Stevenel's blue (del Cerro et al. 1980), and then observed under a light microscope …"

© 2023. Published by The Company of Biologists under the terms of the Creative Commons Attribution License (https://creativecommons.org/licenses/by/4.0/).

RESULTS:

1. Line 168: It is curious to know if there is a regimental number of cells that is required in Phase I before the embryo enters Phase II. Furthermore, after the first unequal cell division, **is there a difference in the activity of the generated apical and basal cell** based on cell division frequency (in generating the column of cells)?
Answer to Rev 2: We observe some plasticity in the number of cells before entry to phase II. It is about 7-9 cells, with an average at 8. Depending on the conditions (e.g. crowding), this number can be higher, up to 10-15. There is no obvious activity of the apical vs basal cells, except that the basal cell seems to be mitotically less active. We are currently studying in depth this phase I and will report about it in another paper. Please note that this phase is the very first step in Saccharina embryogenesis, while our current MS deals with the 2D-3D transition that takes place 15 days later. Therefore, we do not wish to provide detailed description of Phase I in the current MS.

2. Line 170: Since it was mentioned later in the text that polystromatization (division in the Z-plane) proceeds acropetally, do the first longitudinal anticlinal cell divisions (entry to Phase II) also behave acropetally? Basipetally? Or simultaneously?
Answer to Rev 2: Again, a high level of plasticity is applied to the position of these longitudinal anticlinal cell divisions. However, they are usually initiated in the apical half of the embryo. Because it is out of scope of this MS, we did not give details about this step, that will be further described in another paper.

Figure 2: It is suggested for this figure to be presented in a landscape orientation (see Figure 4) to convey “progression” of form from 1D to 3D
Answer to Rev 2:We revised this figure as suggested by Rev 2.

Table 1, Table 2, and Figure 2C, 2G, 2J: The orientation of X- and Y-axes is a bit confusing. **Conventionally, the surface of the growth medium is used as a reference for the X-axis.** Furthermore, kelps grow underwater in an “upright” position (the term “apico-basal” was also mentioned a couple of times) which for the layman will mean the Y-axis.
Answer to Rev 2:We agree with Rev 2 that usually the X-axis is horizontal. However, we found logical to name X-axis the first growth axis, and then the subsequent growth axes Y and Z, as in the geometry field. Therefore, in our study, the first axis is designed regardless of the usual spatial orientation, and it happens that it is the apico-basal, vertical, axis. However, please note that, while in mature specimen this first growth axis is always vertical, in the early embryos it is more frequently with angles ranging from 0 to 90° (i.e.the embryo grow lying down).

Furthermore, the first growth axis in the brown algae Ectocarpus, that grows horizontally, has also been named the X-axis. To be able to compare growth axes in these two algae, we decided to use the same spatial coordinates, regardless of horizontality and verticality. Ectocarpus is an epiphytic alga, whose orientation depends on that of its substratum (usually another, bigger, alga). Therefore, defining the absolute orientation of Ectocarpus filaments in nature is irrelevant.
Figure 2J: There should also be a late-stage diagram where the meristoderm (the actively dividing tissue) is labelled to better visualize the growth points relative to the orientation of the cell division planes).
Answer to Rev 2: Figure 2 is a general figure aiming to establish the vocabulary nomenclature of the following description in the paper. For a 3D spatial representation of the meristoderm, please refer to Fig 9, which is more realistic than Fig 2.

3. Line 256: There is no graph (with statistical tests) comparing morphometric measurements between cell types. There needs to be a visualized quantitation of these metrics aside from scaled diagrams which is usually based on just averages (i.e., means).
Answer to Rev 2: In Fig. S5, we now provide a box plot showing the median, and lower and upper quartile of the distribution of the sizes in the 3 spatial axes of the different cell types in five different tissues (from stipe to young polystroma), together with the result of statistical tests. In addition, the values are given in Table S2, also with the details of the statistical tests.

4. Line 263: Wrong figure citation (?) - might be Suppl. Fig. 4 instead of Suppl. Fig. 2H.
Answer to Rev 2: We acknowledge Rev 2 for spotting this error. In the revised version, most of the figures have been given a different number, and thereby, been checked. We hope that there is no

© 2023. Published by The Company of Biologists under the terms of the Creative Commons Attribution License (https://creativecommons.org/licenses/by/4.0/).

further mistake in this respect.

Figure 7: "Longitudinal and transverse…" – Might be worth clarifying that it is "longitudinal frontal."
Answer to Rev 2: It is actually a longitudinal sagittal section from the stipe. We have edited the legend of this figure.

5. **Line 375**: It is curious to determine how much connections do each cell have at Phase II.
Answer to Rev 2: We are not sure that we understand correctly this remark. Our opinion is that it is interesting to count the number of plasmodesmata to assess the capacity for cell-cell communication. Unfortunately, we could not, as TEM sections are 70nm thick while a cell of Saccharina at that stage is about 15 µm in thickness (Z-axis). Therefore, to be able to estimate the number of plasmodesmata per cell, more than 200 sections of the same embryo must be observed. However, a series of thin sections with straight phase II embryos is still challenging for our means and is anyway out of the general scope of this article. In this paper, our TEM experiments show that at least one pit-field per cell can be observed. This can serve as starting points for more detailed analyses of plasmodesmata distribution at phase II, as it might be suggested by Rev 2.

DISCUSSION:
1. **Line 406**: Not all land plant apical meristems consist of several cell layers. In mosses, there are single cell meristems (simplex meristems) – as is the case in the remaining bryophytes (liverworts and hornworts), some lycophytes and most certainly monilophytes. Please rephrase accordingly. **Answer to Rev 2:** We agree with Rev 2. We have rephrased (lines 404-405): "In contrast, the shoot or root apical meristems of land plants are usually made up of several cell layers (Steeves and Sussex 1989)."

2. **Line 419**: "seemingly making up for lost time." Since the meristoderm is analogous to the population of cells in the apical meristem, it can be argued that this are just multiple apical cells that is dispersed on the superficial layer of the growing sporophyte that actively divides and is responsible for the growth in multiple axes.
Answer to Rev 2: We agree with Rev 2. However, the "seemingly making up for lost time" was referring to the fact that the cells remain undifferentiated up to 15 days after fertilisation. To avoid confusion, we have revised the sentence by removing the "seemingly making up for lost time" (lines 414-418). The comparison with the plant cambium still stands.

3. **Line 434**: Don't use "basal" or "early divergent lineages" to refer to bryophytes. See McDaniel 2021 (10.1111/nph.17241). Also, refrain from anthropomorphization i.e., "struggle"
Answer to Rev 2: We thank Rev 2 for referring us to McDaniel 2021, very informative indeed! Even when working with brown algae, which are *de facto* considered "primitive" organisms, we often fall in the trap of classifying organisms based on datation or morphological complexities. We have revised the text of our MS. (From line 430).

Supplemental Figure 4E, 4F, 4G, 4H: These structures are not clear/obvious in the image. **Brackets is suggested to be used instead of arrows**. Diagrammatic versions of these sections might help in visualizing pit fields and branches.
Answer to Rev 2: We have revised Suppl fig 4 (Fig. S4) as suggested by Rev 2.

Second decision letter

MS ID#: DEVELOP/2022/201519

MS TITLE: Outside in: how kelp embryos shift to 3D growth, an atlas of cell division and differentiation

AUTHORS: Ioannis Theodorou and Benedicte Charrier

© 2023. Published by The Company of Biologists under the terms of the Creative Commons Attribution License (https://creativecommons.org/licenses/by/4.0/).

I have now received the referee's report on the above manuscript, and have reached a decision. The referee's comments are appended below, or you can access them online: please go to BenchPress and click on the 'Manuscripts with Decisions' queue in the Author Area.

The overall evaluation is positive and we would like to publish a revised manuscript in Development, provided that the referee's comments can be satisfactorily addressed. Please attend to all of the reviewer's comments in your revised manuscript and detail them in your point-by-point response. If you do not agree with any of their criticisms or suggestions explain clearly why this is so. If it would be helpful, you are welcome to contact us to discuss your revision in greater detail. Please send us a point-by-point response indicating your plans for addressing the referee's comments, and we will look over this and provide further guidance.

Reviewer 1

Advance summary and potential significance to field

The presence of a meristoderm responsible for the thickness is an interesting statement and is novel. Kelps are under-researched despite their economical and ecological importance.

What the authors claim, in conclusion, is that the inner cells including conductive tissue are derived from an outer meristematic layer of cells: the meristoderm. This is very surprising and strange as this outer region is unprotected. For the case of Dictyota and to a lesser case Dictyopteris (also an unprotected apical meristem, and meristoderm in the case of Dictyopteris), this is not a big issue as these seaweeds just branch adventisiously and use this a vegetative propagation strategy. In kelps with only one blade breakdown of meristemic activity of the meristem would mean end of life, there is only one blade.

Comments for the author

The authors have now added new figures and reworked parts of the text and explained that the authors mean the meristoderm is only responsible for growth in the Z axis (thickness). And that the division in cortex and medulla do occur but are in X-Y. "Our observations combined with the work of Fritsch and Smith led us to propose that in the TZ, all cells divide along the X and the Y axis. However, in the Z axis, mainly the meristoderm cells divide. They are the layer that ensures most of the growth in Z (thickening). That's why we call it a meristodermn and this is the aim of our paper here." Is very a helpful statement. This is diametrically opposed to some statements in previous version for example. "Another remarkable fact of kelp is that all thickening (in the Y-Z plane) comes from the mitotic activity of a single, peripheral cell layer, the meristoderm." Illustrating my level of confusion after the first reading. Now it is clear to me, after having read the rebuttal letter and the figure modifications.

"Imo (in my opinion); The idea that meristoderm cells as the source of new cells divide a tiny cell, which expands into a cortex cell and than would get smaller and elongated and arrange itself in filaments allowing a function as conductive tissue is far fetched. Undoubtedly all cell types here divide in the meristematic region.
Answer to Rev 1: We understand Rev 1 doubts. However, our observations do not support her/his hypothesis. Until it is possible to follow cell division in living organisms and in depth, we think that it will be very difficult to describe in more details, and overall, dynamically, how Saccharina grows in depth."
This is contradicting; there are observations disproving my proposition and the fact that it cannot be tested without invivo observations.
I think you can test and disprove this based on this data, however, see below.

I do not see which observations do outrule this possiblity. It would have been helpful if these would have been specified in the rebuttal. This the alternative hypothesis in the end and disproving this is sort of aim of the paper. It might be the authors interpretation of their observations that does not support this hypothesis.

Instead the authors could test their hypothesis:

© 2023. Published by The Company of Biologists under the terms of the Creative Commons Attribution License (https://creativecommons.org/licenses/by/4.0/).

The authors have added figure S7. “In the revised version, we have added a new Fig. S7 that shows that cells in the TZ divides more periclinally than anticlinally, when compared to the basal part of the blade. In addition to promoting a cylindrical shape, this property also supports a higher abundance of undifferentiated cells in the TZ, and hence, of its “meristematic” nature.

It is this kind of evidence the authors could provide for their claim. However, in this particular case haven’t the authors just showed that at the TZ the outer layer (called here “meristoderm”) is actually multilayered rather than unilayered. This would make a lot of sense and protext the TZ against grazing and provides strengthening. Then the periclinal cell walls are not testimony of cell division and redifferentiation but testimony of double cortex. As happens in many algae in the more basal regiouns (that need to be thicker).
(Here it confusing that the outer layer of Saccharina is called meristoderm, and the more vacualated cells are called cortex (while this is more equivalent to medula of for example Dictyota. But this a strange).

Instead, shouldn’t the evidence lie in for example the 3 layered zone? (Figure 5, step D to step E, in section like Figure 4E and F). The authors have sectioned this zone and must have this data. Here we have 3 cells (and sometimes 4). This is the simplest situation and easiest to understand. 2 outer and one (or sometimes 2) inner. It could be detectable how the cells divide overhere. In what how many cases does it seem to be the medullary cell is dividing and what cases is it the cortical cell that underwent a cell a recent cell divison. In other words is the medulary cell splitting up or are new medullary cells added in the Z axis by division of the outer (“meristodermic”) layer. Wouldn’t this test their hypothesis of the authors in the most simple way?:
Figure 4F seems to suggest to me given the positioning and size of the cells that the 4 layered situation (Figure 4F) is reached by a subdivision of the “cortical” cell. Therefore thickness here is reached by division of the “cortical” cell. Not by the outer layer. Here we should see intermediary situation of small spliced of cells of the outer layer that expand and become medullary like. But my interpretation is off course based on only these two pictures. The authors probably have more.

On the minor comments:
It is very confusing to interpret the blade cells (see Figure S8) and the daughter cells of the outer layer when dividing periclinally “undifferentiated”. This is more semantics, interpretation or convention, rather than pointing to an undifferentiated nature of these cells. They seem very photosynthetically active and need to be differentiated for such a role. Especially the blade cells of course, they make up the thallus and are called here “undifferentiated”.

Table 2 lists Distromatic aand polystromatic for phase II. But Figure 2 shows a monostromatic algae at this stage 2. This is a mistake the authors might want to correct in the final ms?

On the remarks of Red Algae: Yes indeed, I was referring to Delesseriaceae which show resemblance to Laminariaceae. Indeed information is scarce, as is for Laminariaceae as well. Interesting to read Fritsch himself drew the same parallel. But this way of developping is not thàt unique, in view of the existence of the Delesseriaceae.

On the remarkes on Dictyotales: I do not agree with the authors vision on how Dictyotales divide. This is heavily biased on D dichotoma’s way of thickening. But not other species. Rugulopteryx okamurae (D. okomurae) for example has a multilayered medulla at the margins, this occurs more basally. I also read in the mentioned 2020 review on Dictyota (see figure 1 on the left) there are species with a multilayered cortex (or entire medula), but this doubling occurs more basally.
This last thing is very reminiscent to the here presented “meristoderm” activity of the outer layer, producing a multilayered “protective” or “photsynthetic active” layer. At the basal side; a multilayered outer layer is also present in the basal regions and futher thickingen also occurs more basally outside of the “meristematic region”. But this seem not to happen in the well studied simpler type species D. dichotoma.

The rest of the comments were addressed appropriately.

© 2023. Published by The Company of Biologists under the terms of the Creative Commons Attribution License (https://creativecommons.org/licenses/by/4.0/).

Second revision

Author response to reviewers' comments

In black: Reviewer 1
In blue: the authors

Reviewer 1 Advance Summary and Potential Significance to Field:

The presence of a meristoderm responsible for the thickness is an interesting statement and is novel. Kelps are under-researched despite their economical and ecological importance.

What the authors claim, in conclusion, is that the inner cells including conductive tissue are derived from an outer meristematic layer of cells: the meristoderm. This is very surprising and strange as this outer region is unprotected. For the case of Dictyota and to a lesser case Dictyopteris (also an unprotected apical meristem, and meristoderm in the case of Dictyopteris), this is not a big issue as these seaweeds just branch adventisiously and use this a vegetative propagation strategy. In kelps with only one blade breakdown of meristemic activity of the meristem would mean end of life, there is only one blade.

Additional comments from the authors of the MS to the reviewer:
1) There are many examples of meristematic cells that are not protected by a specific tissue. The primary apical meristem of an Angiosperm seedling (e.g. *Arabidopsis*) is not protected against grazing and mechanical damages, when positioned between the two cotyledons.
The apical cell of the moss *Physcomitrium* is unprotected before the first phyllids develops during the gametophytic phase. The ridges of the thallus of the green alga *Coleochaete orbicularis* are directly exposed to the outer environment.
2) In apically growing brown algae like Fucus and *Sargassum*, ablation of the apical meristem promotes the growth of lateral branches (see Moss, New Phytologist 1965) or the establishment of a new meristematic center (see https://www.nature.com/articles/s41598-017-13767-5), and therefore, is also not fatal to these algae. In addition, the sub-apical meristoderm becomes responsible for the emergence of new apical meristematic regions. In kelps, the meristoderm is external and peripheral, similar to the sub-apical regions of apically growing brown algae, but, in contrast, is all around the thallus. Therefore In these algae if a piece of thallus with a peripheral meristoderm is damaged, broken or eaten, the neighbouring regions should compensate for the damage by keeping growing. This was confirmed by our observation in lab conditions.
3) Observations (ours and Katsaros et al., 2021) show that the meristoderm is usually protected by a thick outer cell wall, which surface is covered by a polysaccharide-rich compound commonly referred as a “mucus” by F.E. Fritsch (1945). It is reminiscent of the cuticle covering the thick cell wall of the L1 cell layer in angiosperm aerial tissues.
Altogether, the fact that the meristoderm is a monolayer of cells that ensure all the thickening of an organism is not a trait that would be detrimental for the algae, and does not support Reviewer 1’s concerns.

Reviewer 1 Comments for the Author:

The authors have now added new figures and reworked parts of the text and explained that the authors mean the meristoderm is only responsible for growth in the Z axis (thickness). And that the division in cortex and medulla do occur but are in X-Y. “Our observations combined with the work of Fritsch and Smith led us to propose that in the TZ, all cells divide along the X and the Y axis. However, in the Z axis, mainly the meristoderm cells divide. They are the layer that ensures most of the growth in Z (thickening). That’s why we call it a meristodermn and this is the aim of our paper here.” Is very a helpful statement. This is diametrically opposed to some statements in previous version for example. “Another remarkable fact of kelp is that all thickening (in the Y-Z plane) comes from the mitotic activity of a single, peripheral cell layer, the meristoderm.” Illustrating my level of confusion after the first reading. Now it is clear to me, after having read the rebuttal letter and the figure modifications.

Answer : First, Reviewer’s questions helped us to better describe the thickening process and we are

© 2023. Published by The Company of Biologists under the terms of the Creative Commons Attribution License (https://creativecommons.org/licenses/by/4.0/).

happy that the reviewer finds our additional figures useful. Secondly, please note that the two sentences cited by the reviewer are not completely opposed. The only difference is that now, we expand cell division in Y to cells other than those of the meristoderm only. This mitotic activity is yet at a much lower rate than that of the meristoderm.

“Imo (in my opinion); The idea that meristoderm cells as the source of new cells divide a tiny cell, which expands into a cortex cell and than would get smaller and elongated and arrange itself in filaments allowing a function as conductive tissue is far fetched.

Answer: Reviewer #1 seems to reject the fact that tiny cells resulting from an asymmetrical cell division (ACD) can expand and change shape with time.
1) First, many examples of asymmetrical cell divisions leading subsequently to the formation of large cells are found in the plant literature. In essence many apical meristematic cells (apical meristems) (brown algae, land plants, ferns, see https://www.frontiersin.org/articles/10.3389/fpls.2015.00972/full) divides asymmetrically to give rise to a "tiny" cell that then expands and divides. For example, the guard cells of stomata are formed by several asymmetric cell divisions of mother meristemoid cells and meristemoid cells (https://doi.org/10.3389/fpls.2019.01783). The guard cells are considerably larger than the meristemoid cells. It is also an illustration of self renewal divisions as it occurs in the meristoderm of kelp.
Such process has already been described in brown algae. In *Dictyopteris*, the subsequent "thickening" below the apical meristem at the midrib of each branch results from a meristodermatic-like layer, described by the authors as : «The superficial cell layer of the developing midrib behaves like a meristoderm. Further periclinal divisions add cells inwards which, close to the apex, differentiate into medullary cells and, at more developed thallus regions, into cortical cells (Figs 7, 8). The inner daughter cells of these divisions are smaller than the external ones, which will continue to function as meristodermal cells. In this way, the central region of the thallus becomes multilayered."
(https://www.tandfonline.com/doi/abs/10.1080/00071618800650091). As for Fucales (an order of brown algal species) we suggest a carefull reading of the works from Betty L. Moss, as well as the study on the apical meristoderm of S*argassum muticum* from Linardić and Braybrook (https://www.nature.com/articles/s41598-017-13767-5)
These examples show that the size of a daughter cell of an ACD does not dictate the size of the final derivatives
2) Secondly, regarding the capacity of changes of cell shapes, the cell wall of brown algae is not rigid and fixed in its organisation, but it is flexible and prone to a lot of remodelling (see doi: 10.1016/j.tplants.2018.10.013). Therefore, algal cells can change their shapes, as plant cells do (for example, guard cells of stomata). Transversal section of kelp tissue with medulla very clearly showed an increased deposition of cell wall material (cell wall thickening), while the cell contours have changed from squarrish to round. While turgid cells can expand significantly in response to a softer cell wall, they can also shrink by mechanical compression. Therefore, a soft and constantly remodelled cell wall can result in many cell shape modifications.

Undoubtedly all cell types here divide in the meristematic region.

Answer: We don’t understand what Reviewer N°1 says here. Does he/she mean the meristoderm (peripheral meristem), or the TZ region (longitudinal region with high growth acitivity)? If this is the meristoderm, then all cell types cannot divide in ir because all cell types are not present. They differentiate outside of it, more innerly in the tissue. If the TZ, then in which axes is cell division is considered by the reviewer? Please note that all cell types are observed in all parts of the thallus along the X axis, and not only in the TZ (yet cell types are represented with different abundance in the different regions).

“Answer to Rev 1: We understand Rev 1 doubts. However, our observations do not support her/his hypothesis. Until it is possible to follow cell division in living organisms and in depth, we think that it will be very difficult to describe in more details, and overall, dynamically, how Saccharina grows in depth.”
This is contradicting; there are observations disproving my proposition and the fact that it cannot be tested without invivo observations.
I think you can test and disprove this based on this data, however, see below.

© 2023. Published by The Company of Biologists under the terms of the Creative Commons Attribution License (https://creativecommons.org/licenses/by/4.0/).

I do not see which observations do outrule this possiblity. It would have been helpful if these would have been specified in the rebuttal. This the alternative hypothesis in the end and disproving this is sort of aim of the paper. It might be the authors interpretation of their observations that does not support this hypothesis.

Instead the authors could test their hypothesis:

The authors have added figure S7. “In the revised version, we have added a new Fig. S7 that shows that cells in the TZ divides more periclinally than anticlinally, when compared to the basal part of the blade. In addition to promoting a cylindrical shape, this property also supports a higher abundance of undifferentiated cells in the TZ, and hence, of its “meristematic” nature.
It is this kind of evidence the authors could provide for their claim. However, in this particular case haven’t the authors just showed that at the TZ the outer layer (called here “meristoderm”) is actually multilayered rather than unilayered.

Answer: Only mature specimen have several layers of meristoderm according to Smith. However, in the present MS, we studied the development only of early polystromatic stages.
In these embryos, we did not show that the meristoderm is multilayered in the TZ. We showed that in TZ, the monolayered meristoderm produces by cell division a layer of cells that we named ‘undifferentiated cells”. With our approach, we didn’t observe any mitotic activity for these cells. Therefore we estimate that they differentiate without any division step preceeding cell differentiation. Future microscopy observations on live embryos with appropriate imaging approaches will elucidate the precise behaviour of these cells.
In the paper, we also discussed that this undifferentiated cell layer has a different dynamics in cell differentiation vs cell division compared to the meristodermatic layer. Indeed, in the TZ, the layer of undifferentiated cells results from either faster cell division of the meristoderm cells, or slower cell differentiation of the daugther cells of the divided meristoderm cell. That also accounts for this zone to be named “a meristem” (along the X axis), because of the presence of a layer of undifferentiated cells, in contrast to the other regions of the organism.

This would make a lot of sense and protect the TZ against grazing and provides strengthening.

Answer: How additional cell layers would protect regions of mature thallus that are proned to damage is not the scope of our work and we can only speculate that several layers could indeed help resist against mechanical or enzymatic attacks.
However, many plant organisms have developed strategies to bypass the effect of removal of the apical meristem.
In Fucales (e.g. Moss, New Phytologist 1965, 1970 ; Linardic and Braybrook, Nature Scientific Reports 2017) removal of the apical meristem results in the formation of branches where new apical meristems are formed.
In Laminariales, on the one hand, we cannot find evidence supporting that the removal of part of the meristoderm results in the perishment of the organism. On the other hand, works (https://www.sciencedirect.com/science/article/abs/pii/0022098186902443) show that the TZ, stipe and holdfast regions are generally avoided by grazers. The authors of the above work have sugessted the presence of anti-herbivory compounds against snails at least. When the whole TZ region is removed, the ability to regenerate the blade is lost (https://doi.org/10.1017/S0025315400056071. https://doi.org/10.1007/BF01611380).

Then the periclinal cell walls are not testimony of cell division and redifferentiation but testimony of double cortex.

Answer: We are confused. Previouly, Reviewer 1 talked about a double layer of meristoderm (per half of a tissue section, is it correct?). Now it seems that he/she discusses a double layer of cortex. Maybe Reviewer 1 considers that if two layers of cortex are present, then they differentiate from the two layers of meristoderm?
The thickening of differentiated tissues is not the scope of our paper, as we focused on the emergence of thick tissues from a monolayered, undifferentiated tissue. In the context of very early embryos, we displayed only a single layer of cortex per half of tissue section.

As happens in many algae in the more basal regions (that need to be thicker).

© 2023. Published by The Company of Biologists under the terms of the Creative Commons Attribution License (https://creativecommons.org/licenses/by/4.0/).

Answer: While thick basal regions can be advantageous, this is unrelated with the early developmental stages and the formation of a parenchymatic tissue. As mentioned already, mature kelp species have indeed a meristoderm consisted of several cell layers. The establishment of this multilayered meristoderm or external tissue is not the scope of our work.
(Here it confusing that the outer layer of Saccharina is called meristoderm, and the more vacualated cells are called cortex (while this is more equivalent to medula of for example Dictyota. But this a strange)

Answer: In *Dictyota* species the nomenclature is indeed different but they have different structure than kelps. For instance, the medulla of kelps (innermost tissue of mature organisms) is consisted of sinuous and branched long cells. The medulla of most *Dictyota* species is consisted of conspicuously large transparent cells reminiscent of cortical tissue in kelps. The midrib of *Dictyopteris* (another Dictyotales species) as explained also in the text, has tissue organisation and structure more similar to kelp species. (Compare the histological studies of Smith https://doi.org/10.2307/2436796 with the review of Bogaert on *Dictyota* https://doi.org/10.1007/s10811-020-02121-4 and the work of Katsaros on *Dictyopteris* https://doi.org/10.1080/00071618800650091).

Instead, shouldn't the evidence lie in for example the 3 layered zone? (Figure 5, step D to step E, in section like Figure 4E and F). The authors have sectioned this zone and must have this data. Here we have 3 cells (and sometimes 4). This is the simplest situation and easiest to understand. 2 outer and one (or sometimes 2) inner. It could be detectable how the cells divide overhere. In what how many cases does it seem to be the medullary cell is dividing and what cases is it the cortical cell that underwent a cell a recent cell divison.
In other words is the medulary cell splitting up or are new medullary cells added in the Z axis by division of the outer ("meristodermic") layer. Wouldn't this test their hypothesis of the authors in the most simple way?

Answer: We don't understand what Reviewer 1 is referring to because on Figures 5D to E, and 4E and F, there is no medulla cell that is displayed, but only meristoderm and cortical cells.
Besides, we said in the MS that cortical and medulla cells can divide periclinally. But this event is rare, and the vast majority of cells come from the mitotic activity of the meristoderm. At the end, the cortical and the medulla tissues that can be observed are the sum of these two mitotic activities. The addition of 'new" medulla cells come from the differentiation of the cortical cells mainly, as we described it above (remodelling of the cell wall, change of cell shape, so that cortical cells differentiate little by little into medulla cells).
Additional answers are given below, as the Reviewer continues to raise and discuss this point.

Figure 4F seems to suggest to me given the positioning and size of the cells that the 4 layered situation (Figure 4F) is reached by a subdivision of the "cortical" cell. Therefore thickness here is reached by division of the "cortical" cell. Not by the outer layer. Here we should see intermediary situation of small spliced of cells of the outer layer that expand and become medullary like. But my interpretation is off course based on only these two pictures. The authors probably have more.

Answer: Indeed, we claim that the 4th layer of cells when shifting from Fig 4E to 4F, is not due to a division of the inner cortical cell (there is no medulla cell in this section), but to the division of the "second" (right-hand side) meristoderm layer, as we schematised it in Fig. 5. Our reasons are the following:

1. This is the most parsimonious scenario. The second meristoderm layer is expected to divide as the first one, and to proceed as the first one. How else could it be? Therefore, a symmetrical pattern of cell division and of cell differentiation, with the two outer layers at the origine of it, is the most parsimonious explanation.
2. The photos support this scenario: In Fig S4B, the two layers of cortical cells are separated, while their contours match well those of their respective meristoderm direct neighbours (note that in some locations, the meristoderm cell has already divived in the Y axis, so that two meristoderm cells are observed on the top of only one cortical cell). This is also observed in Fig S4D and Fig S4E, and even more in Fig S2A, where additional layers of cortical cells are produced by the meristoderm monolayer (please focus on the two cortical cells on the right top whose outlines align perfectly with those of the meristoderm cells above, see in the circle of the image below for assistance).

© 2023. Published by The Company of Biologists under the terms of the Creative Commons Attribution License (https://creativecommons.org/licenses/by/4.0/).

This supports that each cortical cell layer is produced by the division of the unique meristoderm cell layer, and not that the second, innermost cortical cell layer is produced from the first, outermost, cortical cell layer (or would it be the other way around?). In Fig S4D, we see small cortical cells (white, big vacuole) in the vicinity of the meristoderm (bottom left), while big ones are more central. The small cortical cells are perfectly aligned with the meristoderm cells (see below image, circle). This supports that these cortical cells are daughter cells of the meristoderm cells as seen above, and that they are younger than the innermost cortical cells. Therefore, the thickening is a centripetal, symmetrical process.

In Fig S4C, the cortical cells are separated by **even more extracellular matrix** (relaxed cell wall made of alginates). In this case, how would it be possible for the layer of one side to produce additional cortical layers for the other side? Therefore, the most simple way is to have always the meristoderm producing cortical cells in a centripetal way.

As anticipated by Reviewer # 1, we have other sections (not shown in the main text, some of them are part of our morphometrical analysis, which will be submitted as raw data) that support this scenario.

We have modified the legend of the figures related to this question. For Fig. 8, we have added (line 690-694): “Note that the contours of the cortical cells in the new polystroma are generally aligned with one or up to three meristodermal cells (A,C-E), which supports that they are derived from periclinal cell division of meristodermal cells (the latter may have divided anticlinally once or twice subsequently, giving 3 meristodermal cell for one cortical cell). No periclinal cell division of cortical cells could be observed in these newly formed and more mature polystromata.”
Legend of Fig S4 has been completed by: “Note the thick cell wall between the two longitudinal layers of C cells, which does not support self-renewal of the cortical tissue by periclinal cell division of C cells. C, D) Pre-medullary cells with protrusions and characteristics of cortical cells (arrows). In D, the large elongated cell appears to be multinucleate. In the bottom left-hand corner, note the progressive differentiation of UC cells into C cells (from top to bottom in the photo). These 4 cells are aligned with the meristodermal cells, confirming that the UC and C cells are derived from meristodermal cells.

On the minor comments:

It is very confusing to interpret the blade cells (see Figure S8) and the daughter cells of the outer layer when dividing periclinally “undifferentiated”. This is more semantics, interpretation or convention, rather than pointing to an undifferentiated nature of these cells. They seem very photosynthetically active and need to be differentiated for such a role. Especially the blade cells of course, they make up the thallus and are called here “undifferentiated”.

Answer: We understand that Reviewer 1 raises the point of the definition of what we named an undifferentiated cell. We think that this is indeed relative to a reference, that is less or more differentiated. For us, “undifferentiated” refers to the cell of Phase II lamina, in which all the cells have a similar shape, mitotic activity and fate (results in preparation for publication): the embryo looks like a monotonous monolayered lamina. Transcriptomics of discrete regions of the lamina would tell whether these cells, that look and behave similarly, are actually similar in terms of gene expression and display no signature for e.g. specific metabolism or signaling pathways as anticipated from their morphology and cell division behaviour. These cells also look like the very initial cells of Phase I embryo. Therefore, up to the stage corresponding to phase III, there is no sign of differentiation in phase II embryos compared to phase I.
However, we agree with Reviewer 1 that they have chloroplasts as indicated in Table 2, and must be able to perform photosynthesis (all cells look the same and must then provide carbohydrate for the growth of the embryo). This is not incompatible with the fact that these cells are less differentiated than the cells present in Phase III lamina, that have a different intracellular organisation (large vacuole for the cortical cells) and different cell shapes (cylindrical for the medulla cells, close to spherical for the cortical cells, cuboid for the meristoderm cells) and larger size (cortical cells and medulla cells). Therefore, we maintain the choice of our semantics.

In the present revised MS, we justified our choice by adding in lines 196-200: “We consider the cells

© 2023. Published by The Company of Biologists under the terms of the Creative Commons Attribution License (https://creativecommons.org/licenses/by/4.0/).

of phase I and phase II embryos to be 'undifferentiated' because, although photosynthetically active, they share a similar regular cuboidal shape, relatively low mitotic activity and a similar pattern of symmetrical cell division. As a result, the embryo resembles a monotonous monolayer lamina." Similarly, as we also use this term for one of the daughter cell of the meristoderm (i.e. UC cell), we have added lines 268-269: "We have called this inner daughter cell "undifferentiated" because it corresponds to an intermediate stage before differentiation." This was also said in lines 302-305 of the previous revised version: "Finally, in all regions of the polystromatic blade, a few undifferentiated cells can be observed, seemingly the result of asymmetrical cell divisions of meristoderm cells (green cells schematised in Fig. 6 and seen in Fig. 8B,E-I), which eventually differentiate into cortical cells (Fig. 6B,C and Fig. 7C)."

Table 2 lists Distromatic aand polystromatic for phase II. But Figure 2 shows a monostromatic algae at this stage 2. This is a mistake the authors might want to correct in the final ms?
Answer: We thank Reviewer 1 for spotting this mistake. Instead of "Phase II", we meant "Early Phase III", and instead of "Phase III", we meant "Late Phase III". "Phase I" should be "Phase II". This has been corrected in the revised version of Table 2. We also added a line, corresponding to the undifferentiated cells observed in the TZ. We omitted this cell type in the previous version of the MS.

On the remarks of Red Algae: Yes indeed, I was referring to Delesseriaceae which show resemblance to Laminariaceae. Indeed information is scarce, as is for Laminariaceae as well. Interesting to read Fritsch himself drew the same parallel. But this way of developping is not thàt unique, in view of the existence of the Delesseriaceae.

Answer : We didn't suggest that Fritsch drew the same parallel. On the contrary, in Fritsch's book there is a clear distinction between parenchymatic species (kelps) and pseudoparenchymatic species (most species of red macroalgae), which we are not sure to what extent it is functional or informative. However, as we already tried to explain in the previous rebuttal letter, the emergence of additional cell layers in Delesseriaceae species is unknown and there are no image data in the present literature from which we can draw any conclusion for resemblance with the mechanism described in Saccharina. In the absence of any data or literature that can support a resemblance, we cannot safely come to the conclusion that polystromatisation in the two groups, i) Saccharina-like kelps and ii) Delesseriaceae from red algae is similar.

On the remarks on Dictyotales: I do not agree with the authors vision on how Dictyotales divide. This is heavily biased on *D dichotoma*'s way of thickening. But not other species. Rugulopteryx okamurae (D. okomurae) for example has a multilayered medulla at the margins, this occurs more basally. I also read in the mentioned 2020 review on Dictyota (see figure 1 on the left) there are species with a multilayered cortex (or entire medula), but this doubling occurs more basally. This last thing is very reminiscent to the here presented "meristoderm" activity of the outer layer, producing a multilayered "protective" or "photsynthetic active" layer. At the basal side; a multilayered outer layer is also present in the basal regions and futher thickingen also occurs more basally outside of the "meristematic region". But this seem not to happen in the well studied simpler type species D. dichotoma.

Answer: We thank Reviewer 1 for sharing his/her knowledge with us. We did not claim that Dictyota does not thicken : lines 400- 401: "Growth in only peripheral cells is common to other brown algae, such as the Fucales *Fucus* and *Sargassum* or in the Dictyotales *Dictyota dichotoma*, in which only one or two apical cells ensure all growth in all three axes (Katsaros and Galatis 1985; Bogaert et al. 2020) ".
We claim that *Dictyota dichotoma* does not have a meristoderm and the 3D growth is established at the premises of its apical meristem. However, to be more correct and to refer to the well studied organism, we have added the species name to avoid confusion in the legend of Fig 1 and in line 400-401 in the revised version.

The rest of the comments were addressed appropriately.

© 2023. Published by The Company of Biologists under the terms of the Creative Commons Attribution License (https://creativecommons.org/licenses/by/4.0/).

Third decision letter

MS ID#: DEVELOP/2022/201519

MS TITLE: Outside in: how kelp embryos shift to 3D growth, an atlas of cell division and differentiation

AUTHORS: Ioannis Theodorou and Benedicte Charrier
ARTICLE TYPE: Research Article

I am happy to tell you that your manuscript has been accepted for publication in Development, pending our standard ethics checks.

Reviewer 1

Advance summary and potential significance to field

The primary focus of this paper centers on the proposal that cell division in the Z-axis primarily occurs by division of “meristoderm/epidermal” cells. Given the paper's significance and the unconventional notion that reliance on a meristematic layer at the outside for growth may be surprising, particularly in light of herbivory concerns or in the context of thickening in other seaweeds, one would anticipate the provision of quantitative evidence to support this intriguing hypothesis. Regrettably, such evidence appears to be lacking in my assessment, as I was unable to locate it upon a careful reexamination of the manuscript.

Only in this rebuttal letter do the authors reference Figure S4 of the supplement, which – indeed - seem to suggest that the daughter cells of the "meristoderm" are actually undergoing a process of redifferentiation into cortex cells.

My contention is whether this interpretation is adequately substantiated with quantitative evidence. Currently, it remains an unsupported assertion based on the interpretation of certain sections. The data, particularly in Figure 5, should provide a basis for their proposed model, especially regarding the transition from 3 to 4 layers.

The authors put forth a model in which the absence of epidermal cells would preclude an influx of cortical cells. However, it is plausible that cortical cells undergo division independently, with an additional influx of daughter cells of epidermal/”meristoderm” cells that subsequently end up in the cortical niche and redifferentiate in a manner into cortical cells. However this process might occur at a slow pace, it is difficult to quantitate this based on sections.

Comments for the author

Additional comments where noted with >
>I will focus solely on the relevant section, acknowledging that there are other intriguing aspects where the authors present valid points or with which I may disagree. However, delving into these matters would distract from the main topic.

Instead, shouldn’t the evidence lie in for example the 3 layered zone? (Figure 5, step D to step E, in section like Figure 4E and F). The authors have sectioned this zone and must have this data. Here we have 3 cells (and sometimes 4). This is the simplest situation and easiest to understand. 2 outer and one (or sometimes 2) inner. It could be detectable how the cells divide overhere. In what how many cases does it seem to be the medullary cell is dividing and what cases is it the cortical cell that underwent a cell a recent cell divison.

In other words is the medulary cell splitting up or are new medullary cells added in the Z axis by division of the outer (“meristodermic”) layer. Wouldn’t this test their hypothesis of the authors in the most simple way?

© 2023. Published by The Company of Biologists under the terms of the Creative Commons Attribution License (https://creativecommons.org/licenses/by/4.0/).

Answer: We don't understand what Reviewer 1 is referring to because on Figures 5D to E, and 4E and F, there is no medulla cell that is displayed, but only meristoderm and cortical cells.
Besides, we said in the MS that cortical and medulla cells can divide periclinally. But this event is rare, and the vast majority of cells come from the mitotic activity of the meristoderm. At the end, the cortical and the medulla tissues that can be observed are the sum of these two mitotic activities. The addition of 'new" medulla cells come from the differentiation of the cortical cells mainly, as we described it above (remodelling of the cell wall, change of cell shape, so that cortical cells differentiate little by little into medulla cells).

Additional answers are given below, as the Reviewer continues to raise and discuss this point.

Figure 4F seems to suggest to me given the positioning and size of the cells that the 4 layered situation (Figure 4F) is reached by a subdivision of the "cortical" cell. Therefore thickness here is reached by division of the "cortical" cell. Not by the outer layer. Here we should see intermediary situation of small spliced of cells of the outer layer that expand and become medullary like. But my interpretation is off course based on only these two pictures. The authors probably have more.

Answer: Indeed, we claim that the 4th layer of cells when shifting from Fig 4E to 4F, is not due to a division of the inner cortical cell (there is no medulla cell in this section), but to the division of the "second" (right-hand side) meristoderm layer, as we schematised it in Fig. 5. Our reasons are the following:

1. This is the most parsimonious scenario. The second meristoderm layer is expected to divide as the first one, and to proceed as the first one. How else could it be? Therefore, a symmetrical pattern of cell division and of cell differentiation, with the two outer layers at the origine of it, is the most parsimonious explanation.
>The alternative scenario is that the cortical cell (yes apologies– I got confused with medula) is dividing symmetrically producing 2 cortical cell layers. This seems the case if on has to believe the cell contours in this 1 picture.
So how else could it be?: by periclinal division of the cortical cell.
The cell linings seem to be consistent with cell division of the cortical cell.
>If the claim of the authors is right there should be many transition zones with small spliced of cells (undifferentiated cells). Instead if the scenario of symmetric cell division of the cortical cells producing 2 cortical layers when the cells are big enough, is true. We most of the time this symmetric 2 layers and not 1 smaller and 1 bigger cortical layer.

2. The photos support this scenario: In Fig S4B, the two layers of cortical cells are separated, while their contours match well those of their respective meristoderm direct neighbours (note that in some locations, the meristoderm cell has already divived in the Y axis, so that two meristoderm cells are observed on the top of only one cortical cell). This is also observed in Fig S4D and Fig S4E, and even more in Fig S2A, where additional layers of cortical cells are produced by the meristoderm monolayer (please focus on the two cortical cells on the right top whose outlines align perfectly with those of the meristoderm cells above, see in the circle of the image below for assistance).
This supports that each cortical cell layer is produced by the division of the unique meristoderm cell layer, and not that the second, innermost cortical cell layer is produced from the first, outermost, cortical cell layer (or would it be the other way around?). In Fig S4D, we see small cortical cells (white, big vacuole) in the vicinity of the meristoderm (bottom left), while big ones are more central. The small cortical cells are perfectly aligned with the meristoderm cells (see below image, circle). This supports that these cortical cells are daughter cells of the meristoderm cells as seen above, and that they are younger than the innermost cortical cells. Therefore, the thickening is a centripetal, symmetrical process.

In Fig S4C, the cortical cells are separated by even more extracellular matrix (relaxed cell wall made of alginates). In this case, how would it be possible for the layer of one side to produce additional cortical layers for the other side? Therefore, the most simple way is to have always the meristoderm producing cortical cells in a centripetal way.
> Here it is true that this picture in supplement (S4C) suggest that daughter cells producted by cell division of the "meristoderm/epidermal" cells are becoming hyaline and cortex like. It therefore could be that these cells getting in the niche of the cortex are redifferentiating into a cortexlike

© 2023. Published by The Company of Biologists under the terms of the Creative Commons Attribution License (https://creativecommons.org/licenses/by/4.0/).

morphology. But it is difficult to make predictions on the speed they do this with. But indeed it is some evidence that some thickness is provided by the “meristoderm/epidermal” layer, as the authors propose. Which is interesting.
Howevery, one could still assume that all cell types (present at the 3 – 5 thick stage: meristemal and cortex cells) are dividing and perhaps that most of the thickness is generated by the division of the cortex cells.
Additionally the medulla fillaments are dividing from the cortex cells so these are not produced by the epidermal cells as well (figure S4C). So this thickness is not generated by the “meristoderm”, unless – indeed – if they were all direct descendent daughter cells of the “meristoderm”/epidermis. But this is not the case if both cortex and epidermal cells produce cortex cells.

© 2023. Published by The Company of Biologists under the terms of the Creative Commons Attribution License (https://creativecommons.org/licenses/by/4.0/).
